# Indian Summer Monsoon Rainfall: Implications of Contrasting Trends in the Spatial Variability of Means and Extremes

**DOI:** 10.1371/journal.pone.0158670

**Published:** 2016-07-27

**Authors:** Subimal Ghosh, H. Vittal, Tarul Sharma, Subhankar Karmakar, K. S. Kasiviswanathan, Y. Dhanesh, K. P. Sudheer, S. S. Gunthe

**Affiliations:** 1 Dept. of Civil Engineering, Indian Institute of Technology Bombay, Mumbai 400076, India; 2 Interdisciplinary Program in Climate Studies, Indian Institute of Technology Bombay, Mumbai 400 076, India; 3 Centre for Environmental Science and Engineering, Indian Institute of Technology Bombay, Mumbai 400 076, India; 4 EWRE Division, Dept. of Civil Engineering, Indian Institute of Technology Madras, Chennai, India; Institute of Botany, CHINA

## Abstract

India’s agricultural output, economy, and societal well-being are strappingly dependent on the stability of summer monsoon rainfall, its variability and extremes. Spatial aggregate of intensity and frequency of extreme rainfall events over Central India are significantly increasing, while at local scale they are spatially non-uniform with increasing spatial variability. The reasons behind such increase in spatial variability of extremes are poorly understood and the trends in mean monsoon rainfall have been greatly overlooked. Here, by using multi-decadal gridded daily rainfall data over entire India, we show that the trend in spatial variability of mean monsoon rainfall is decreasing as exactly opposite to that of extremes. The spatial variability of extremes is attributed to the spatial variability of the convective rainfall component. Contrarily, the decrease in spatial variability of the mean rainfall over India poses a pertinent research question on the applicability of large scale inter-basin water transfer by river inter-linking to address the spatial variability of available water in India. We found a significant decrease in the monsoon rainfall over major water surplus river basins in India. Hydrological simulations using a Variable Infiltration Capacity (VIC) model also revealed that the water yield in surplus river basins is decreasing but it is increasing in deficit basins. These findings contradict the traditional notion of dry areas becoming drier and wet areas becoming wetter in response to climate change in India. This result also calls for a re-evaluation of planning for river inter-linking to supply water from surplus to deficit river basins.

## Introduction

Indian summer monsoon rainfall (ISMR) over India contributes approximately 80% of the annual precipitation during June-September (Jun–Sep) [1]. India’s agricultural output, economy, and societal well being depend significantly on the stability, variability and extremes of summer monsoon rainfall [1,2]. Deviations in the ISMR from the long-term mean may profoundly impact the agricultural productivity and Gross Domestic Product (GDP). Understanding the impacts of climate change on the ISMR and changes in its temporal and spatial patterns poses a key research challenge [3] with significant implications on water resources and management policies [4,5]. Water resource management for climate change adaptation is challenging not only because of the uncertainty associated with multi-model ensembles [6], scenarios [7] and reanalysis products [8] but also due to disagreements [9] within various analyses and explanations for temporal trends in the mean and extremes. These disagreements primarily arise from an incomplete understanding of monsoon processes and dynamics and differences in the selection of spatial extent and resolutions [9]. Policy-related decisions regarding regional water resources and management depend on the variability of the ISMR and require regional hydro-meteorological information. However, the regional hydro-climatic analyses of the ISMR have been poorly addressed partly because state-of-the-art general circulation models cannot simulate the regional distribution of the monsoon [3] and because land surface feedback, the amount of recycled precipitation [10], changes in the Land Use Land Cover (LULC) are poorly understood. Understanding the trend and pattern of the spatial variability of ISMR [11] significantly impacts large-scale water distribution and can influence the debatable initiative of nationwide inter-basin water transfer [12,13] intended to supply water from surplus to deficit river basins. The spatial aggregation of intensity and frequency of extreme rainfall events over Central India are reported to be significantly increasing [14], whereas they are spatially non-uniform with increasing spatial variability at the local scale [9,15,16]. The reasons behind this increase in the spatial variability of extremes are poorly understood, and the trends in the spatial variability of the mean monsoon rainfall have been greatly overlooked. Here, we analyze the long-term trend in the spatial variability of ISMR and its detailed policy and management implications. In the present study, we elucidate trends in the spatial variability of the mean and extremes of the Indian monsoon rainfall. The trends and patterns of water availability with their spatial variability are important inputs for major nationwide decisions, such as the interlinking of river basins, which has major ecological implications [17] and is consequently considered to be highly contentious in policy-making. Notably, this study describes trends in the spatial variability of rainfall and consequently differs from all previously reported studies, in which trends in the quantity of rainfall were analyzed. Trends in the ISMR describe changes in the total water availability in India, but they are not useful at the river basin scale of water management due to the spatial heterogeneity of monsoon rainfall. Conversely, trends in the spatial variability describe the changes in the spatial distribution of water availability, which are important for water management at the river basin scale and to plan large-scale river interlinking.

## Data and Methods

The gridded daily rainfall data (mm/day) from the India Meteorological Department (IMD), for the years 1901–2004 at a resolution of 1° [18] were used to compute the gridded seasonal mean and extremes. The rainfall data were generated with the interpolation method proposed by Shepard [19] from a fixed network of 1384 stations that had a minimum data availability of 70% to minimize the risk of generating temporal inhomogeneity. These data product has been previously used in many Indian monsoon studies [20,21]. The convective and stratiform rainfall data were obtained from a high resolution (0.5° × 0.667°) reanalysis dataset, i.e., from a Modern-Era Retrospective analysis of the Research and Application (MERRA) database [22]. The rainfall from June to September was temporally averaged to compute the seasonal mean, whereas the block maxima method of the extreme value theory was implemented to characterize the extreme rainfall. The seasonal (June to September) maxima was first computed and then fitted to the Generalized Extreme Value (GEV) distribution [9,23,24]. The goodness-of-fit was evaluated using the Kolmogorov-Smirnov (KS) test. If the KS test failed, we fit a nonparametric Gaussian kernel distribution [20], which has been found suitable for estimating extreme quantiles [25]. Rainfall with a non-exceedance probability of 0.98 in the Cumulative Distribution Function (CDF) corresponds to a 50-year return period, which is considered ‘extreme’ in the present study. We computed the spatial variability of both the mean and extremes with a 30-year moving window throughout the entire time period. For extremes, the 50-year return levels were computed for each 30-year window, and the spatial variability was then computed. Furthermore, the trends in the spatial variability at a 5% significance level were computed with a Modified Kendall approach [26], which considers both linear and nonlinear trends and is applicable to autocorrelated data (S1 Text**)**. The hydrologic simulations were performed with a Variable Infiltration Capacity (VIC) model [27] in energy balance mode by embedding a snow band formulation, for which the maximum temperature, minimum temperature, rainfall and wind velocity were considered as input meteorological variables (S1 Text**)**. The model was validated with satellite-based observation from the European Space Agency-Climate Change Initiative (ESA-CCI) soil moisture data [28,29]. The water yield was first computed at the grid level by adding the surface runoff and base flow. They were further spatially averaged for major river basins in India.

## Results and Discussion

**Fig 1:**
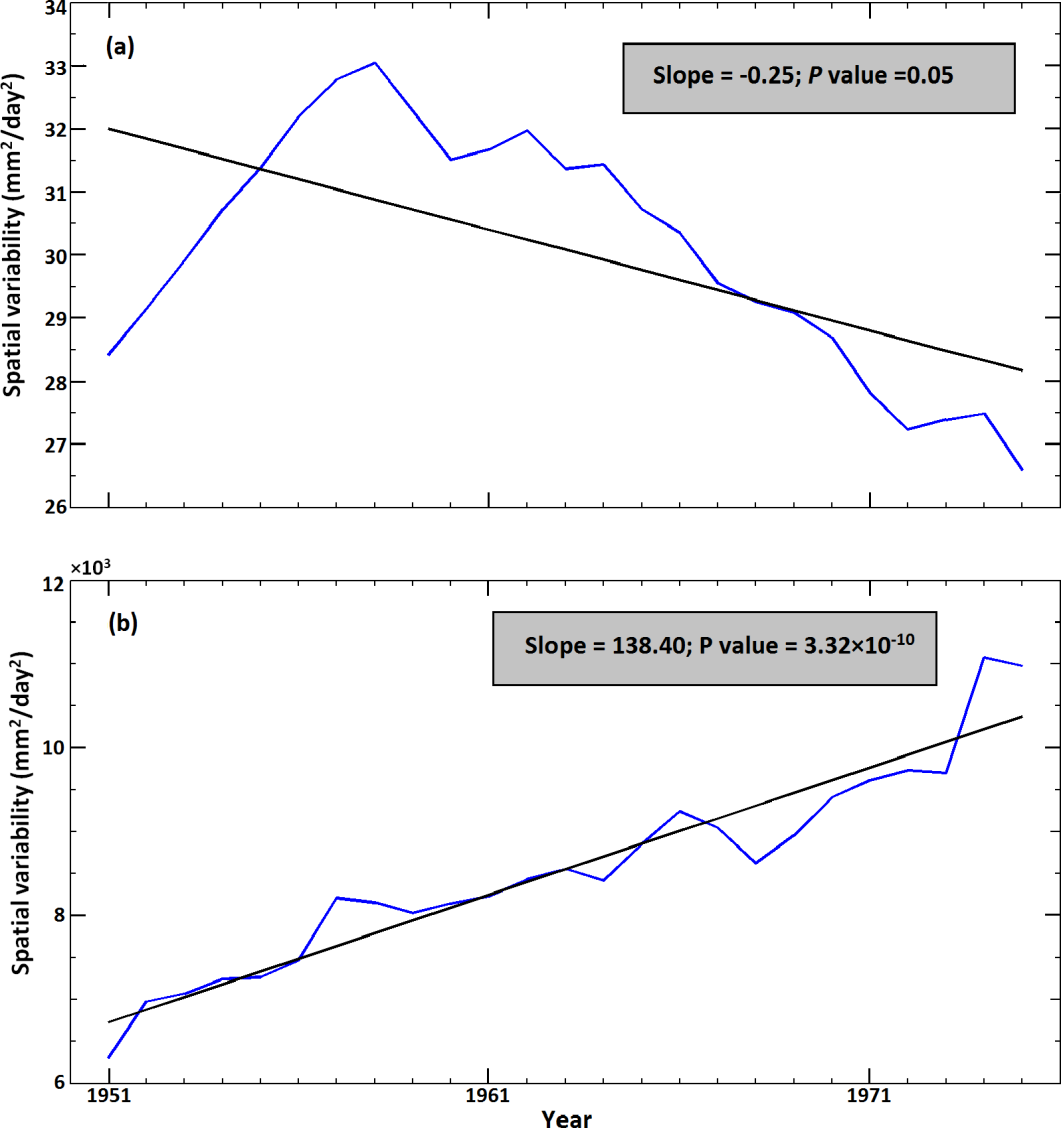
Trends in the spatial variability of the ISMR from 1951 to 2004 over the Indian region. (a) Trend in the spatial variability of the mean monsoonal rainfall over the Indian region with a 30-year overlapping moving window. The blue line indicates the spatial variability of the mean rainfall, whereas the black solid line shows the fitted trend (linear) line. The mentioned trend value was computed with the modified Mann-Kendall approach. (b) Trend in the spatial variability of the extreme rainfall (corresponding to a 50-year return period) with the blue and black lines representing analyses similar to those shown in panel (a). The trends in both panels are significant at the 5% significance level.

The spatial variability of the mean and extreme of the ISMR exhibit opposing trends. In the present case, rainfall associated with a return period of 50 years (S1 Text) was considered an extreme. Trends were calculated using the modified Mann-Kendall approach [26]. The spatial variability of the mean ISMR exhibits a marked and significant decrease, whereas the spatial variability of extremes exhibits a significant increase (Fig 1) at 5% significance level. Similar result is also observed with the product of finer resolution rainfall for longer duration (S2 Fig). Such strong contrasting trends in spatial variability throughout India in the mean and extreme ISMR constitute a significant challenge for policy makers in regional water management. This also poses two immediate and pertinent research questions: (i) the applicability of large scale inter-basin water transfer by river inter-linking to address the spatial variability of water availability in India, especially when the spatial variability of the mean ISMR is decreasing and (ii) the reasons underlying the opposing patterns in the spatial variability of the mean and extremes.

**Fig 2:**
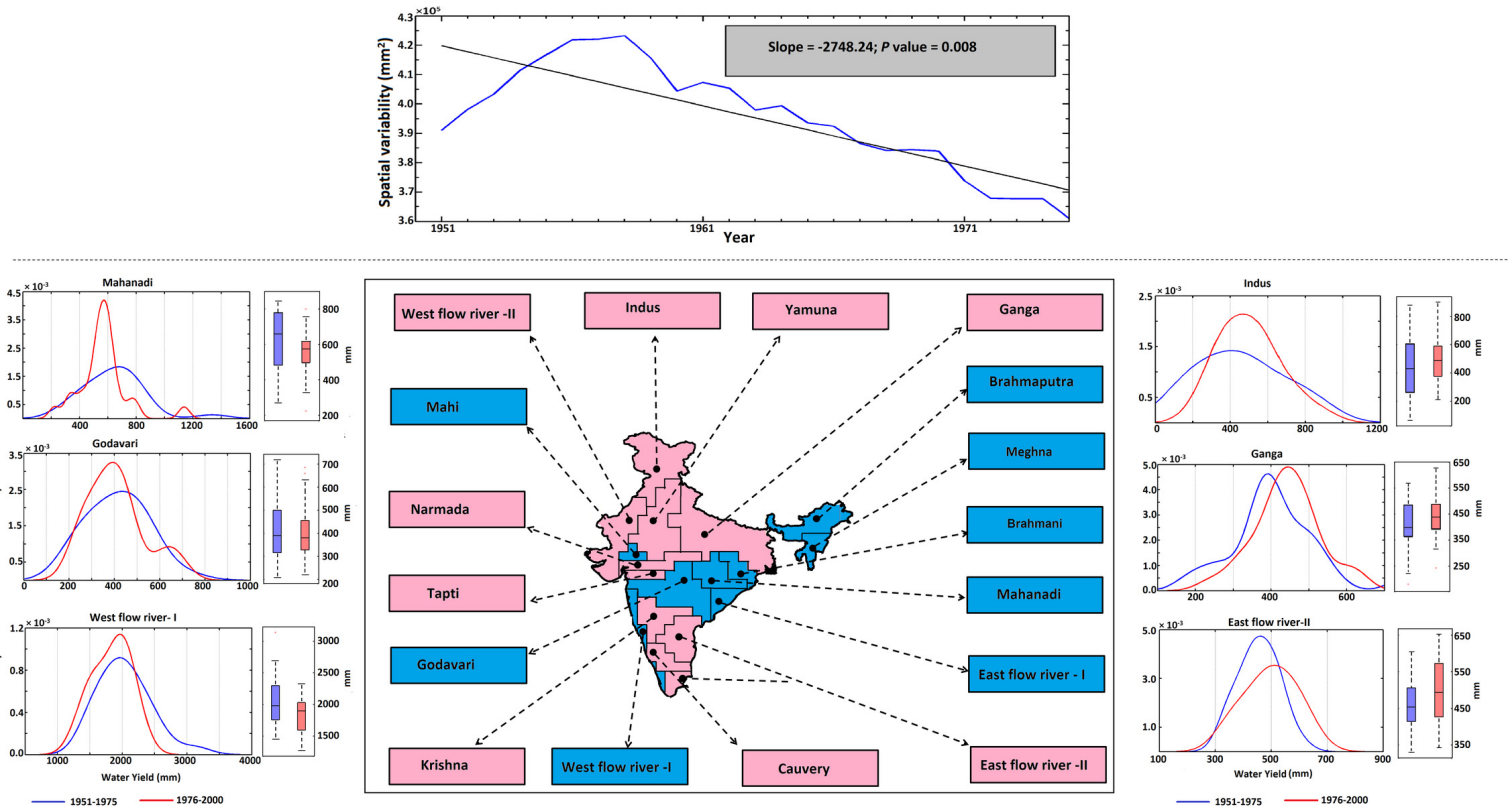
The trend in the water yield over the entire Indian region and the changing pattern of the same for selected surplus and deficit river basins. The panel in the upper part (separated by a dotted line) indicates the spatial variability of the water yield (blue) for all basins combined over the Indian region from 1951 to 2004 with a 30-year overlapping moving window. The black solid line shows the fitted trend (linear). The modified Mann-Kendall trend was observed to be significant at the 5% level. The map in the lower part of the figure depicts the surplus (blue) and deficit (magenta) water basins of India. The names of the rivers belonging to surplus/deficit basins are indicated in the respective boxes. The panels around the map are exemplary graphs showing the changes in water yield during the recent period (1976–2000) compared with the past (1951–1975) for six major rivers belonging to surplus (Mahanadi, Godavari and West flow river I) and deficit (Indus, Ganga and East flow river–II) water basins. The blue and red curves represent the PDFs of the water yield for the past and recent periods, respectively. The shift of the red curve in the positive direction with respect to the blue curve indicates the increase in water yield and vice versa. The water yields are also presented in terms of a box-whisker plot, with red and blue boxes representing the recent and past periods, respectively.

We address the first point with hydrological simulations over entire India and by computing the water yield [30] (sum of the surface runoff and base-flow) in the major river basins. The observed streamflow data were excluded from the analysis because they are considered as “classified” for major river basins in North India (e.g., Ganga) [31]. Moreover, the available datasets may be affected by control structures built at different times, which bias the trend, and these data may consequently not be useful to assess the time evolution of hydrological scenarios in India. Instead, we used the simulated results for all of India obtained from the Variable Infiltration Capacity (VIC) model [27,32], which is widely used for global hydrological studies [33,34]. The performance of the VIC model was assessed and validated with soil moisture satellite data (S3 Fig). The results of the VIC-simulated soil moisture and the observed soil moisture were consistent, both in terms of the correlation (at 5% significance level) and the nRMSE from 1979–2004. The VIC-simulated soil moisture is positively correlated and the nRMSE values were less than 20% in most part of the country. The surplus and deficit river water basins were identified (Fig 2, lower panel) based on water yield, population and water demand (including agricultural demand), as detailed by Gupta and Deshpande [35]. The spatial variability in the gridded water yield over the entire India exhibited a significant decreasing trend (Fig 2, upper panel), which is consistent with the decreasing spatial variability of mean ISMR. Since the spatial variability of water yield is mainly dependent on the spatial variability of rainfall, evapotranspiration (ET), soil moisture, and temperature, we computed the trends and association between these parameters (S4 Fig). While the warming is significant over India (S4A Fig), the simulated ET and soil moisture showed significant decreasing trend (S4B and S4C Fig), which is attributed to the drying of ISMR [36,37]. In addition to this, we observed poor association between temperature and ET over India (S4D Fig). Similar association was reported in the literature [38], where the station level observed ET was poorly correlated with the surface temperature. It has also been reported [39], that the trends and pattern of ET are dominated by the available soil moisture (which is largely controlled by the precipitation) rather than changes in the temperature. Hence, the changes in the water yield is largely contributed by the changes in the ISMR. The analyses with the changes in water yields for different basins show, the water yields in major deficit river basins, such as, Indus, Ganga and East Flow River–II, have increased in recent years (1976–2000) compared with previous years (1951–1975). The changes are visible in terms of shifts in the Probability Density Functions (PDFs) and differences in the percentiles of box-whisker plots (Fig 2, lower panel). Increases of more than 10% were observed for several descriptive statistics, such as the mean, 25th percentile, median and 75th percentile (Table A in S1 Text). The remaining deficit basins either exhibit very small changes (within 10%) or they are very small basins in terms of area coverage. The only exception is the Krishna River basin. The water yields of major surplus basins, such as Mahanadi, Godavari and West Flow River–I, have exhibited decreases in recent periods. The water yields show decreases of more than 10% for the Mahanadi and West Flow River–I. For other surplus basins, the changes are within 10%.

**Fig 3:**
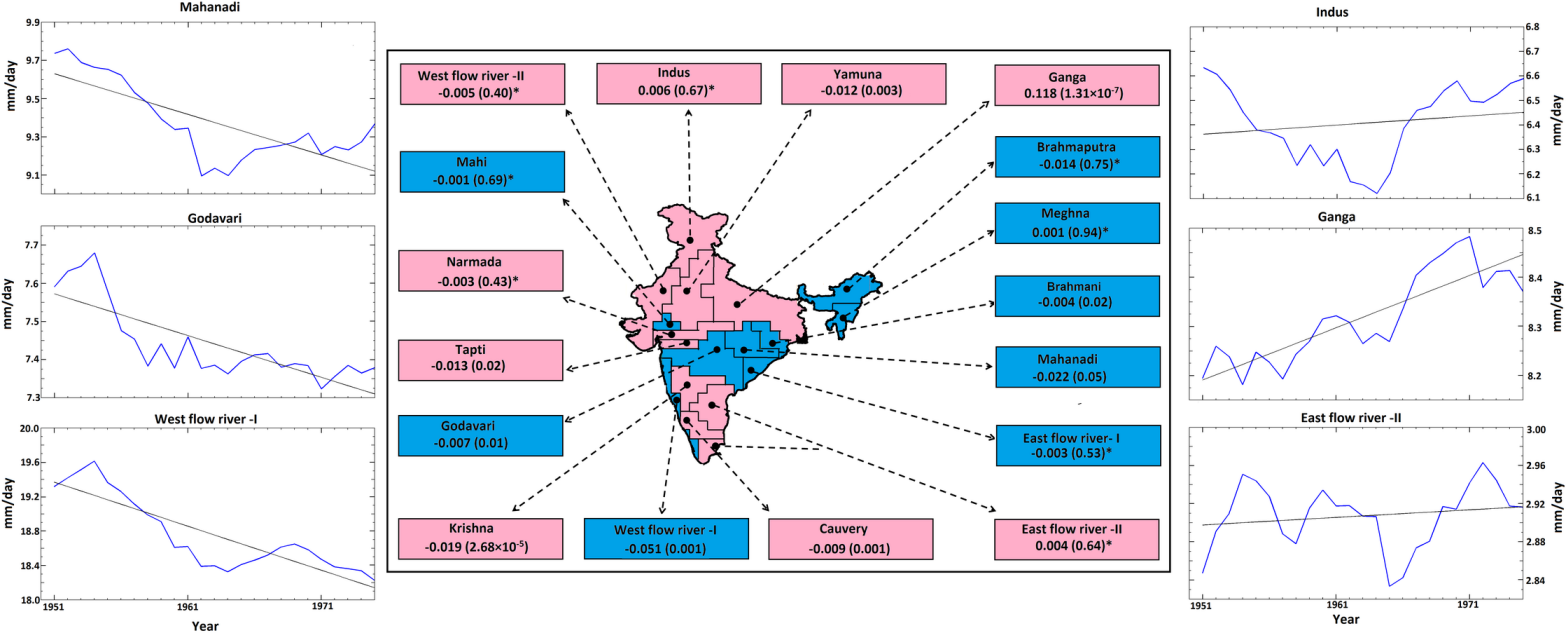
The trend in precipitation for selected rivers in surplus and deficit water basins. The map depicts the surplus (blue) and deficit (magenta) water basins of India. The names of rivers belonging to surplus/deficit basins are indicated in the respective boxes. The values in the boxes are the magnitude (p value) of the trend estimated by the modified Mann-Kendall approach, where a star (*) indicates that the trend is insignificant at the 5% level. The panels around the map are exemplary graphs showing the trends for four major rivers belonging to surplus (Mahanadi, Godavari and West flow river -I) and deficit (Indus, Ganga and East flow river II) water basins. The black solid line shows the fitted trend (linear). The trend is observed to be significant at the 5% level for surplus basins (decreasing); however, for deficit basins, it is significant only for the Ganga basin (increasing).

**Fig 4:**
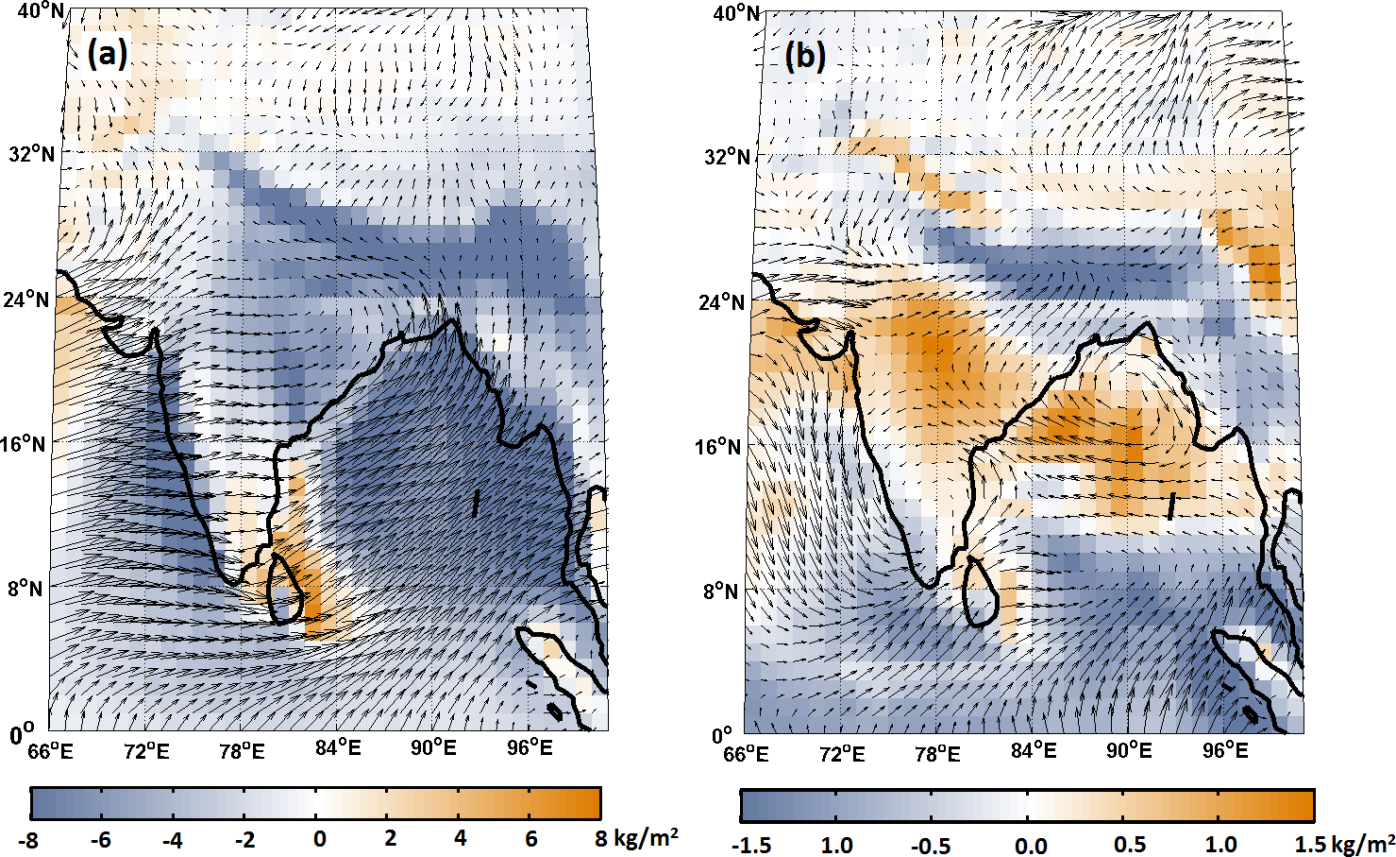
Vertically integrated moisture flux (VIMF) and wind patterns over India for monsoon season. (a) Mean VIMF and wind patterns during 1951–2004. (b) Changes in the mean VIMF and wind patterns during the two periods (1976–2000 and 1950–1975). The positive (negative) value of VIMF exhibits divergence (convergence) over the study region.

To reconfirm our conclusions, we conducted a trend analysis of the ISMR over water surplus and deficit river basins in India (Fig 3). The major surplus basins, such as the Mahanadi, Godavari, West Flow River–I, and Brahmani, exhibit significant decreases in rainfall. The other surplus basins (except Meghna) also exhibited decreases in rainfall, but these changes were not significant at the 95% confidence level. The major water deficit basin, Ganga, exhibits significant increases in rainfall, whereas the Yamuna, Krishna and Cauvery basin exhibit decreases. The decrease in the monsoon rainfall in the surplus river basins, which are majorly present in the core Indian monsoon zone, may be due to the drying of rainfall in these region during recent decades [36,37]. We address this issue by analyzing the changes in the mean vertically integrated moisture flux (VIMF) and the wind patterns over Indian monsoon region. We use ERA 20C reanalysis VIMF and wind data product from 1951–2000. The monsoon over Indian region is typically associated with a strengthened cyclonic circulation, with the moisture flux converging over this region (Fig 4A). However, when the changes in mean vertically integrated moisture flux (VIMF) and wind patterns are analyzed (Fig 4B), we found an anticyclonic circulation leading to divergence in VIMF, especially in the central part of India, along with convergence in the Gangetic plains. Hence, this could be the reason for which the major surplus basins have a decreasing rainfall trend whereas the Ganga basin exhibited increasing rainfall trend. Further, the decreases in rainfall in the water surplus basins do not concur with the notion that wet areas are becoming wetter and dry areas are becoming drier due to climate change [40,41]. Though, some of the recent literature [42,43] argued that, the hypothesis of "wet gets wetter and dry gets drier" is not valid in all parts of the globe, however, testing of this hypothesis over India have been greatly overlooked. The results from our analysis confirm that, the paradigm “wet gets wetter and dry gets drier” is not valid for the Indian scenario. Our analysis also raises concerns about the suitability of major nation-wide projects related to river water-basin interlinking, in which the sustainability of water surplus conditions in river basins in response to a changing climate is not ascertained. Therefore, the water demand in a surplus basin first needs to be assessed and met under decreasing water availability scenarios before transferring water to the deficit basins. Hence, we argue that planning for inter-basin water transfer necessitates an immediate reassessment with a systematic approach.

**Fig 5:**
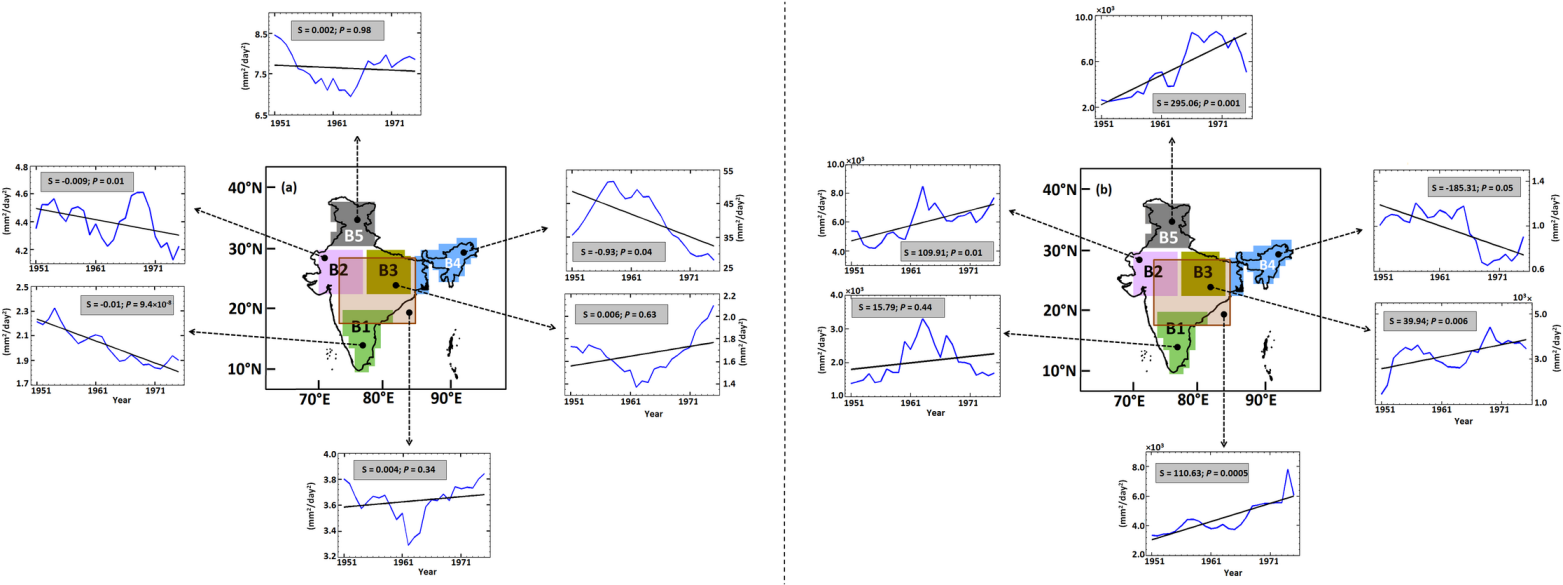
Trends in the spatial variability of the ISMR from 1951 to 2004 over the five different regions with a 30-year overlapping moving window. The graphs associated with map (a) indicated by the dotted arrow demonstrate the trend in the spatial variability of the mean monsoonal rainfall (blue), with the black line indicating the fitted trend (linear) line. The mentioned trend values were computed with the Modified Mann-Kendall approach. Trends are significant only for regions B1, B2 and B4. The graphs associated with map (b) are similar to those of (a) except for the extreme rainfall corresponding to a 50-year return period; specifically, the trend is significant for all regions except region B1. The transparent box in both maps (a and b) indicates the central Indian region (74.5°E to 86.5°E and 16.5°N to 26.5°N) as adopted by G06, wherein the trend in the spatial variability of the mean and extreme monsoonal rainfall is insignificant and significant, respectively. Please note that the scales of the ordinate are different for all graphs associated with both maps. Refer to the S1 Text for more details about the selection of the regions (B1-B5).

**Fig 6:**
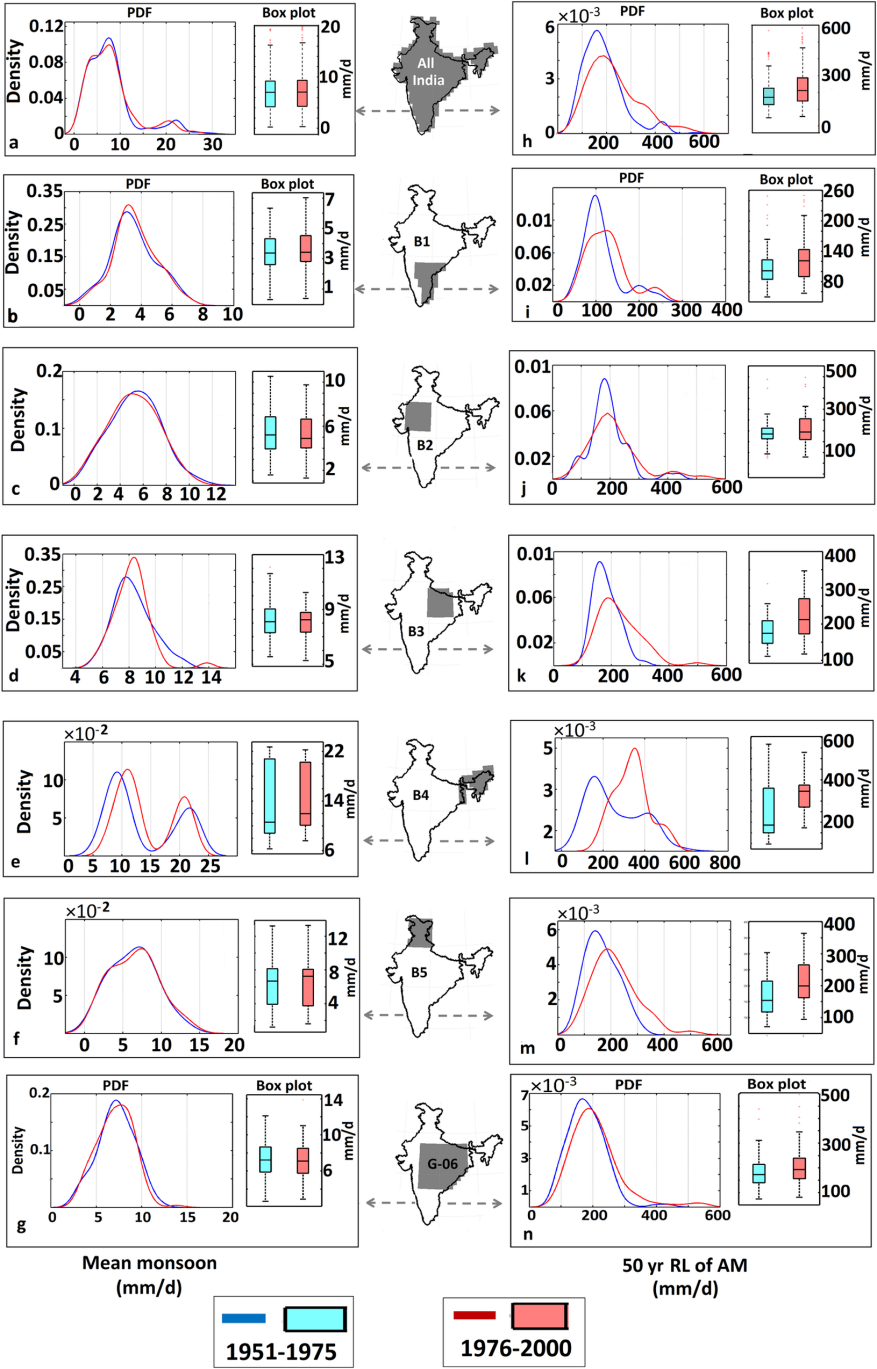
PDFs and box plots showing the spatial variation of the mean (a-g) and extreme rainfall (h-n) during two periods (1950–1975 and 1976–2000). For extremes throughout India, B2, B3 and B5 exhibit increases in the spatial variability resulting from increases in the sparseness and decreases in kurtosis of the PDFs. The box plot also shows an increase in the interquartile range (IQR) for these boxes, indicating an increase in the spatial variability. For the mean monsoon, no significant differences are observed between two time periods.

To investigate the systematic nature and consistency of the opposing trends in the spatial variability of the mean and extreme ISMRs, we selected five regions of equal grid-size from different agro-climatic zones (Table B in S1 Text): B1 –Peninsular India (leeward side of the Western Ghats), B2 –Western India (NW India), B3 –Central India (Gangetic Basin), B4 –North East India (foothill of Eastern Himalayas), and B5 –North India (Fig 5). The trends in the spatial variability of the mean and extreme ISMRs significantly differed among these regions. B1, B2 and B4 exhibit significant decreases at 5% significance level in the spatial variability of the mean monsoon, whereas the remaining two regions exhibit insignificant trends. For extremes, the B2, B3, and B5 regions show clear and significant increases in spatial variability at 5% significance level. Importantly, the Central Indian region was assumed to be homogeneous by Goswami et al. [14] (referred hereafter as GO6) but exhibits a significant increase in the spatial variability of extremes. This interpretation is confirmed by a detailed statistical analysis of two separate time periods (1950–1975 and 1976–2000) that indicates the difference in the spatial variability of the mean and extremes with box-whisker plots and PDFs (Fig 6). Significant differences in the width of PDFs and heights of whiskers, which were only observed for extremes for all the regions, indicate strong increases in the spatial variability of extremes and identify this region as inhomogeneous. However, for mean, the changes in the PDF's are not significant for all the regions for two time periods.

We explain the contrasting nature of trends in the spatial variability of the mean and extreme ISMR by qualitatively analyzing the relative contribution of convective and stratiform rainfall. In general, local scale processes largely govern the convective rainfall, whereas stratiform rainfall originates from large-scale processes [44]. For this analysis, we use the satellite-retrieved rainfall data by the Tropical Rain Measuring Mission (TRMM-PR) [44,45] from 1998 to 2013; specifically, the fraction of convective rainfall to total monsoon rainfall was computed. The rainfall in Northern and Central India appears to be dominated by convective precipitation, whereas stratiform precipitation is dominant over Southern Peninsular India (S5A Fig), an area that did not exhibit a clear and significant trend in the spatial variability of extremes. Furthermore, we performed an analysis similar to the Modern-Era Retrospective Analysis for Research and Application (MERRA) reanalysis data starting from 1981 to substantiate these results, which showed significant positive correlation with IMD rainfall data at 5% significance level (S6 Fig). Although the trend in the spatial variability is insignificant for stratiform rainfall (S5B Fig), the spatial variability of rainfall resulting from convective precipitation exhibits clear and significant increase over the entire Indian region at 5% significance level (S5C Fig). Based on these trends, we hypothesize that the increase in the spatial variability of extremes in North and Central India emanates from convective precipitation. As evident in S5A Fig, regions in which stratiform rainfall is predominant do not show increases in the spatial variability of extremes. Trends in the spatial variability obtained from longer-term reanalyses, such as the European Reanalysis 20th Century (ERA-20C) [46], are similar to those of MERRA (S7 Fig); however, the spatial variability of convective rainfall for JRA-55 (a Japan reanalysis product) [47] does not exhibit significant changes. This finding may be due to the coarser resolution of this reanalysis product, which cannot simulate the finer details required for local-scale convective rainfall.

The spatial variability of stratiform rainfall does not exhibit significant trends, and the reason underlying the decrease in the spatial variability of the mean ISMR consequently remains unexplained and requires hypothesis-driven model studies, which is considered a potential scope and direction for future research. However, the spatial variability of extremes is strongly increasing as a result of convective precipitation, which is caused by a combination of local-scale geophysical processes, most likely perturbations in the cloud properties due to increased aerosols [48], land surface feedback via precipitation recycling [10], urbanization [20; 49], deforestation or land use and land cover (LULU) changes. However, testing the sensitivity analysis of these processes to the spatial variability of extreme rainfall needs regional models run and may be considered as a scope of future research. Furthermore, we show that the spatial variability of extreme rainfall in India is very high, and large-scale studies consequently do not significantly enhance our understanding of scientific processes and projections (S8 Fig). For example, the correlations between individual grid and the spatial regional mean as well as the respective trends in the correlations computed for regions B1 –B5 show significantly high gridded correlations for the mean but low to moderate correlations for extremes. For region G06, the correlation pattern for the mean indicates a significantly high correlation over the entire region, whereas the correlation for extremes is very low for the majority of grids, with insignificant trends in the respective correlations (S9 Fig). This finding raises important questions regarding the validity of rainfall extreme analyses over large spatial regions, especially for India. Furthermore, the postulation in selecting spatially homogenous Central India for analyzing temporal trends in rainfall extremes appears to be ambiguous because the spatial homogeneity in the total mean monsoon rainfall does not resemble relative extremes (S9 Fig). We also found that the changes in the spatial variability of the extreme monsoon rainfall were exorbitantly higher than that of its mean, and the changes in the spatial variability of the mean and extremes are contrasting for all the regions except B5 (Table C in S1 Text).

## Summary

We observed that the spatial variabilities of the mean and extreme ISMR exhibit clear opposing and significant trends over India, thus highlighting the need for more informed hydrologic impact assessment followed by water resource planning and management. The decrease in the spatial variability of the total monsoon rainfall observed in response to decreases in precipitation for surplus basins bears important policy implications and warrants the reconsideration of the ambitious development of inter-basin water transfer planning via river linking, which not only has ecological and financial implications but also may not be sustainable. The decreases (increases) in the surplus (deficit) river basins strongly contradict the traditional notion of wet (dry) areas becoming wetter (drier) over India. Our conclusion corroborates a study conducted by Greve et al. [42], who reported that the paradigm of dry areas becoming drier and wet area becoming wetter is invalid over most of the global land area, including India. Furthermore, we caution that the reasons underlying the decrease in the spatial variation of the mean remain unidentified and require hypothesis-driven model and process studies. Scientific interpretations of the extremes of ISMR warrant additional detailed validation, given the considerable spatial variability due to the dominance of convective rainfall. Our conclusions should benefit national-scale climate-water adaptation and regional preparedness, and their root causes should be identified by future follow-up research activities.

## Supporting Information

S1 Fig Trend in the spatial variability of the mean and extremes computed with a data resolution of 1°. The spatial variability of the mean does not exhibit a trend pre-1950 (a) and a significant decrease post-1950 (b). The spatial variability of extremes does not exhibit a trend pre-1950 (c) and a significant increase post-1950 (d).
(TIF)

S2 Fig Trend in the spatial variability of the ISMR for the period 1901–2013 computed with 0.25° resolution rainfall data over the Indian region with a 30-year overlapping moving window. (a) The spatial variability of the mean monsoonal rainfall (blue), with the black solid line indicating the fitted trend line, and (b) the spatial variability for rainfall extremes (blue) with the black line representing the linear trend. The respective Mann-Kendall trend and associated p values are individually presented for each panel.
(TIF)

S3 Fig Validation of VIC outputs. (a) Correlation between the simulated and observed soil moisture from 1979–2004. (b) Normalized Root Mean Square Error (nRMSE) for the same period.
(TIF)

S4 Fig Evapotranspiration (ET) trend and its association with surface temperature over India during monsoon season. Trends in temperature (a), ET (b) and soil moisture (c) over India during 1951–2004, respectively. (d) shows a scatter plot of ET and temperature over India. The values inset the figures represent magnitude of the correlation (R) and slope for the respective variables. All the values are significant at 10% significance level.
(TIF)

S5 Fig The stratiform and convective precipitation over all of India as derived from TRMM-PR with a spatial resolution of 0.5° and MERRA database with a spatial resolution of 0.5°× 0.667° for the years 1998–2013 and 1981–2005, respectively. (a) Ratio of convective to total precipitation obtained from TRMM for all of India. (b) Trend in the spatial variability of the Indian Summer Monsoon Rainfall (ISMR; June-September) for the stratiform precipitation obtained from MERRA database over the Indian region (blue), where the black line indicates the fitted trend (linear) line. The Modified Mann-Kendall trend is insignificant at the 5% level. (c) is similar to (b) except for the trend in convective precipitation, which is significant at the 5% level. The spatial data obtained from TRMM and MERRA were re-gridded to IMD at a 1° resolution using a linearly interpolated technique.
(TIF)

S6 Fig Correlation between MERRA and IMD precipitation dataset. (TIF)

S7 Fig Trends in the spatial variability for convective and stratiform precipitation over the Indian region during the Monsoon with ERA-20C and JRA-55 data. (a) The trend in the spatial variability of convective precipitation (blue) during 1951–2004 for ERA-20c reanalysis data, where the black line indicates the fitted trend (linear) line. The modified Mann-Kendall trend is significant at the 5% level. (b) is similar to (a) but shows stratiform precipitation, for which the trend is insignificant at the 5% level. Trends in convective precipitation (c) and stratiform precipitation (d) during 1961–2004 for JRA-55 reanalysis data. The modified Mann-Kendall trend is insignificant at the 5% level for both convective and stratiform precipitation. The spatial data obtained from ERA-20c and JRA-55 were regridded to a 1° resolution (same as the IMD data resolution) using a linearly interpolated technique.
(TIF)

S8 Fig Correlation between the mean of the given region and individual grids belonging to the same region and their respective trends. (a) Spatial correlation for the monsoonal mean and (b) spatial correlation for the seasonal maxima. (c) The trend in the spatial correlation of individual grid points over the given region estimated with a 30-year overlapping moving window for the monsoonal mean. (d) same as (c) but for seasonal maxima.
(TIF)

S9 Fig Same as Figure S3 but for the central Indian region (74.5°E to 86.5°E and 16.5°N to 26.5°N) as adopted by Goswami et al. (2006). (TIF)

S1 Text Supplementary Information. Contains complete description of methods and also the Tables.
(DOCX)

## References

[1] Gadgil S, Gadgil S. The Indian Monsoon, GDP and agriculture. Econ Polit Weekly. 2006 11 25; 41(47): 4887–4895.

[2] Webster PJ, Magana VO, Palmer TN, Shukla J, Tomas RA, Yanai MU, et al Monsoons: Processes, predictability, and the prospects for prediction. J Geophys Res. 1998 6 28; 103(C7):14451–510.

[3] Turner AG, Annamalai H. Climate change and the South Asian summer monsoon. Nat Clim Chang. 2012 8 1; 2(8): 587–95.

[4] Mall RK, Gupta A, Singh R, Singh RS, Rathore LS. Water resources and climate change: an Indian perspective. Curr Sci. 2006 6 25;90(12):1610–26.

[5] Archer DR, Forsythe N, Fowler HJ, Shah SM. Sustainability of water resources management in the Indus Basin under changing climatic and socio economic conditions. Hydrology and Earth System Sciences. 2010 8 27;14(8):1669–80.

[6] Stainforth DA, Aina T, Christensen C, Collins M, Faull N, Frame DJ, et al Uncertainty in predictions of the climate response to rising levels of greenhouse gases. Nature. 2005 1 27;433(7024):403–406. 10.1038/nature03301 15674288

[7] Ghosh S, Mujumdar PP. Nonparametric methods for modeling GCM and scenario uncertainty in drought assessment. Water Resour Res. 2007 7 1;43(7). 10.1029/2006wr005351

[8] Kannan S, Ghosh S, Mishra V, Salvi K. Uncertainty resulting from multiple data usage in statistical downscaling. Geophys Res Lett. 2014 6 16;41(11):4013–4019. 10.1002/2014GL060089

[9] Ghosh S, Das D, Kao SC, Ganguly AR. Lack of uniform trends but increasing spatial variability in observed Indian rainfall extremes. Nat Clim Chang. 2012 2 1;2(2):86–91.

[10] Pathak A, Ghosh S, Kumar P. Precipitation Recycling in the Indian Subcontinent during Summer Monsoon. Journal of Hydrometeorology. 2014 10;15(5):2050–2066. 10.1175/jhm-d-13-0172.1

[11] Konwar M, Parekh A, Goswami BN. Dynamics of east‐west asymmetry of Indian summer monsoon rainfall trends in recent decades. Geophys Res Lett. 2012 5 28;39(10). 1–6. 10.1029/2012GL052018

[12] Misra AK, Saxena A, Yaduvanshi M, Mishra A, Bhadauriya Y, Thakur A. Proposed river-linking project of India: a boon or bane to nature. Environmental Geology. 2007 2 1;51(8):1361–76.

[13] Rao BP, Rao PV, Jaisankar G, Amminedu E, Satyakumar M, Rao PK. Interlinking of River Basins: A Mega Harvesting Plan-A. J. Ind. Geophys. Union. 2010 1;14(1):31–46.

[14] Goswami BN, Venugopal V, Sengupta D, Madhusoodanan MS, Xavier PK. Increasing trend of extreme rain events over India in a warming environment. Science. 2006 12 1;314(5804):1442–1445. 17138899 10.1126/science.1132027

[15] Ghosh S, Luniya V, Gupta A. Trend analysis of Indian summer monsoon rainfall at different spatial scales. Atmospheric Science Letters. 2009 10 1;10(4):285–90. 10.1002/asl.235

[16] Krishnamurthy CK, Lall U, Kwon HH. Changing frequency and intensity of rainfall extremes over India from 1951 to 2003. J Clim. 2009 9;22(18):4737–4746. 10.1175/2009jcli2896.1

[17] Lakra WS, Sarkar UK, Dubey VK, Sani R, Pandey A. River inter linking in India: status, issues, prospects and implications on aquatic ecosystems and freshwater fish diversity. Rev. Fish Biol. Fish. 2011 9 1;21(3):463–79.

[18] Rajeevan M, Bhate J, Jaswal AK. Analysis of variability and trends of extreme rainfall events over India using 104 years of gridded daily rainfall data. Geophys Res Lett. 2008 9 1;35(18). L18707 10.1029/2008GL035143

[19] Shepard D. A two-dimensional interpolation function for irregularly-spaced data. InProceedings of the 1968 23rd ACM national conference 1968 Jan 1 (pp. 517–524). ACM.

[20] Vittal H, Karmakar S, Ghosh S. Diametric changes in trends and patterns of extreme rainfall over India from pre‐1950 to post‐1950. Geophys Res Lett. 2013 6 28;40(12):3253–3258. 10.1002/grl.50631

[21] Joshi MK, Rai A. Combined interplay of the Atlantic multidecadal oscillation and the interdecadal Pacific oscillation on rainfall and its extremes over Indian subcontinent. Clim Dyn. 2014 9;44(11–12):3339–3359.

[22] Rienecker MM, Suarez MJ, Gelaro R, Todling R, Bacmeister J, Liu E, et al MERRA: NASA's modern-era retrospective analysis for research and applications. J Clim. 2011 7;24(14):3624–3648.

[23] Coles S. An introduction to statistical modeling of extreme values London: Springer; 2001 12.

[24] Katz RW, Parlange MB, Naveau P. Statistics of extremes in hydrology. Adv Water Resour. 2002 12 31;25(8):1287–1304.

[25] Vittal H, Singh J, Kumar P, Karmakar S. A framework for multivariate data-based at-site flood frequency analysis: Essentiality of the conjugal application of parametric and nonparametric approaches. J Hydrol (Amst). 2015 6 30;525:658–675.

[26] Hamed KH, Rao AR. A modified Mann-Kendall trend test for autocorrelated data. J Hydrol (Amst). 1998 1 30;204(1):182–196.

[27] Liang X, Lettenmaier DP, Wood EF, Burges SJ. A simple hydrologically based model of land surface water and energy fluxes for general circulation models. J Geophys Res. 1994 7 20;99: 14415–14418.

[28] Dorigo W, Jeu R, Chung D, Parinussa R, Liu Y, Wagner W, et al Evaluating global trends (1988–2010) in harmonized multi‐satellite surface soil moisture. Geophys Res Lett. 2012 Sep 1;39(18). 10.1029/2012GL052988

[29] Dorigo WA, Gruber A, De Jeu RA, Wagner W, Stacke T, Loew A, et al Evaluation of the ESA CCI soil moisture product using ground-based observations. Remote Sens Environ. 2015 6 1;162:380–395.

[30] Niu J, Sivakumar B, Chen J. Impacts of increased CO 2 on the hydrologic response over the Xijiang (West River) basin, South China. J Hydrol (Amst). 2013 11 15;505:218–227.

[31] Jain SK. Ganga Basin Response. Current Science. 2014 3 10;106(5):652–3.

[32] Cherkauer KA, Bowling LC, Lettenmaier DP. Variable infiltration capacity cold land process model updates. Glob Planet Change. 2003 7 31;38(1):151–159.

[33] Barnett TP, Pierce DW, Hidalgo HG, Bonfils C, Santer BD, Das T, et al Human-induced changes in the hydrology of the western United States. science. 2008 2 22;319(5866):1080–1083. 10.1126/science.1152538 18239088

[34] Painter TH, Deems JS, Belnap J, Hamlet AF, Landry CC, Udall B. Response of Colorado River runoff to dust radiative forcing in snow. Proc Natl Acad Sci U S A. 2010 10 5;107(40):17125–17130. 10.1073/pnas.0913139107 20855581 PMC2951423

[35] Gupta SK, Deshpande RD. Water for India in 2050: first-order assessment of available options. Curr Sci. 2004 5 10;86(9):1216–1224.

[36] Saha A, Ghosh S, Sahana AS, Rao EP. Failure of CMIP5 climate models in simulating post-1950 decreasing trend of Indian monsoon. Geophys Res Lett. 2014 10 28;41(20):7323–30.

[37] Roxy MK, Ritika K, Terray P, Murtugudde R, Ashok K, Goswami BN. Drying of Indian subcontinent by rapid Indian Ocean warming and a weakening land-sea thermal gradient. Nature communications. 2015 6 16;6. 10.1038/ncomms8423 26077934

[38] Padmakumari B, Jaswal AK, Goswami BN. Decrease in evaporation over the Indian monsoon region: implication on regional hydrological cycle. Climatic change. 2013 12 1;121(4):787–99.

[39] Ramarao MV, Krishnan R, Sanjay J, Sabin TP. Understanding land surface response to changing South Asian monsoon in a warming climate. Earth Syst Dyn Discuss. 2015 1 1;6:1–34.

[40] Huang P, Xie SP, Hu K, Huang G, Huang R. Patterns of the seasonal response of tropical rainfall to global warming. Nat Geosci. 2013 5 1;6(5):357–361.

[41] Biasutti M. Climate change: Future rise in rain inequality. Nat Geosci. 2013 5 1;6(5):337–338. 10.1038/ngeo1814

[42] Greve P, Orlowsky B, Mueller B, Sheffield J, Reichstein M, Seneviratne SI. Global assessment of trends in wetting and drying over land. Nat Geosci. 2014 10 1;7(10):716–721. 10.1038/ngeo2247

[43] Feng H, Zhang M. Global land moisture trends: drier in dry and wetter in wet over land. Scientific reports. 2015;5. 10.1038/srep18018 PMC4676011 26658146

[44] Pokhrel S, Sikka DR. Variability of the TRMM-PR total and convective and stratiform rain fractions over the Indian region during the summer monsoon. Clim Dyn. 2013 7 1;41(1):21–44. 10.1007/s00382-012-1502-1

[45] Choudhury AD, Krishnan R. Dynamical response of the South Asian monsoon trough to latent heating from stratiform and convective precipitation. Journal of the Atmospheric Sciences. 2011 6;68(6):1347–1363. 10.1175/2011jas3705.1

[46] Poli P, Hersbach H, Tan D, Dee D, Thepaut JN, Simmons A, et al. The data assimilation system and initial performance evaluation of the ECMWF pilot reanalysis of the 20th-century assimilating surface observations only (ERA-20C). 2013.

[47] Kobayashi S, Ota Y, Harada Y, Ebita A, Moriya M, Onoda H, et al The JRA-55 reanalysis: General specifications and basic characteristics. Journal of the Meteorological Society of Japan. 2015;93(1):5–48. 10.2151/jmsj.2015-001

[48] Lau KM, Kim KM. Observational relationships between aerosol and Asian monsoon rainfall, and circulation. Geophys Res Lett. 2006 11 1;33(21). 10.1029/2006GL027546

[49] Shastri H, Paul S, Ghosh S, Karmakar S. Impacts of urbanization on Indian summer monsoon rainfall extremes. J Geophys Res Atmos. 2015 1 27;120(2):496–516.

