# Supplementary figures and images for: Indian Summer Monsoon Rainfall: Implications of Contrasting Trends in the Spatial Variability of Means and Extremes
Source: PLoS One. 2016 Jul 27;11(7):e0158670. doi: 10.1371/journal.pone.0158670 (PMC4963102; doi:10.1371/journal.pone.0158670)

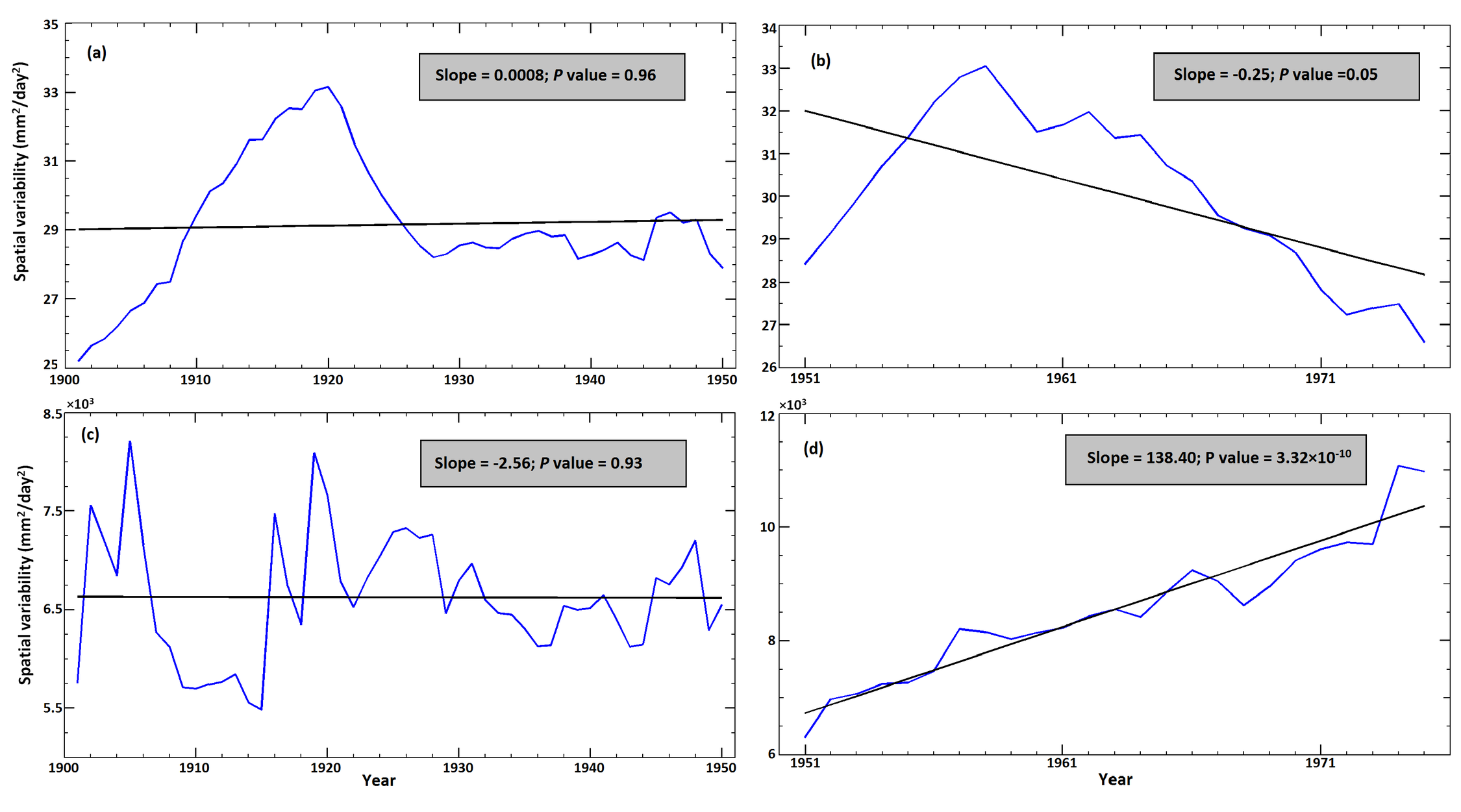

Supplement: S1 Fig — The spatial variability of the mean does not exhibit a trend pre-1950 (a) and a significant decrease post-1950 (b). The spatial variability of extremes does not exhibit a trend pre-1950 (c) and a significant increase post-1950 (d). (TIF) [file pone.0158670.s001.tif]

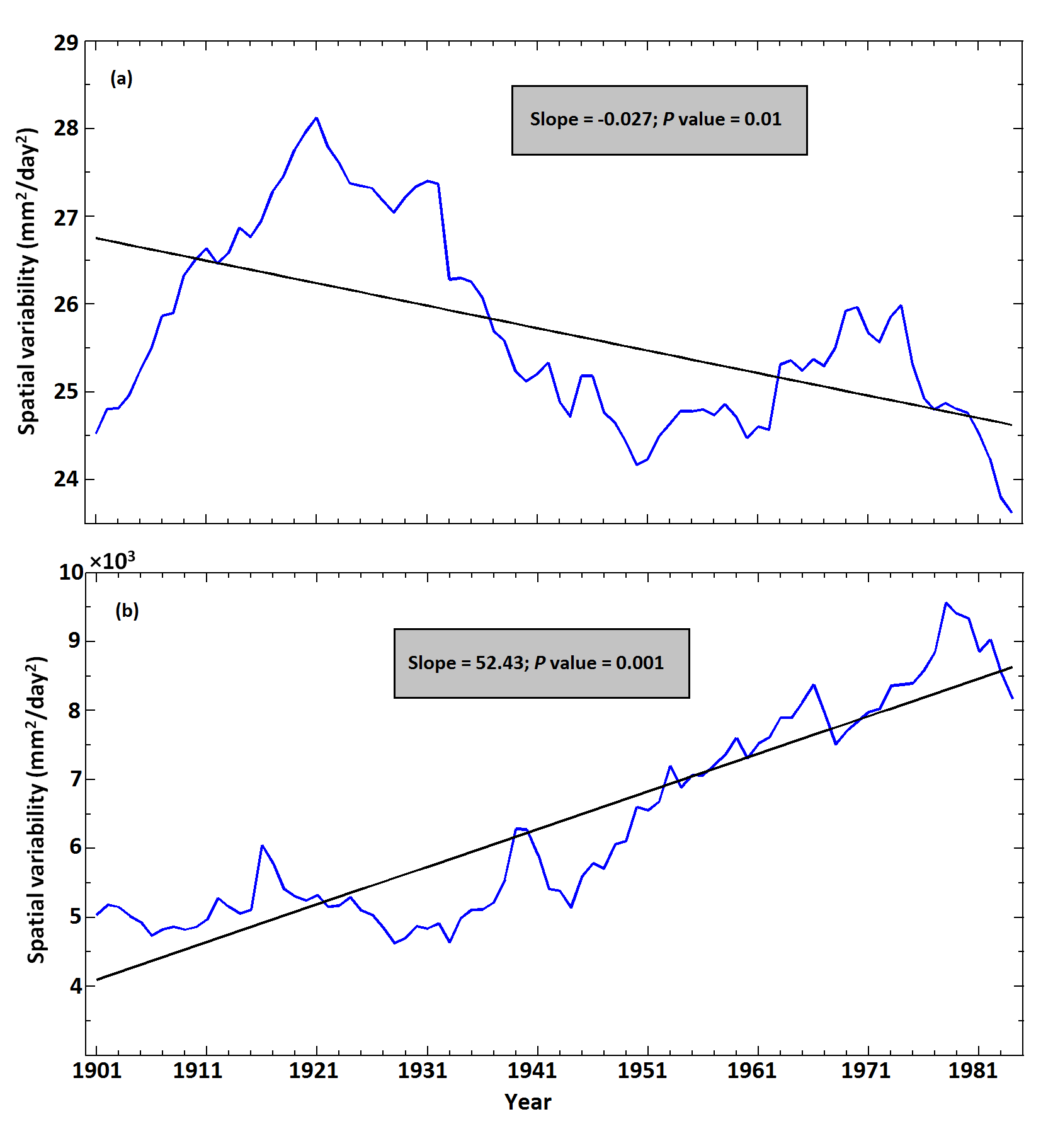

Supplement: S2 Fig — (a) The spatial variability of the mean monsoonal rainfall (blue), with the black solid line indicating the fitted trend line, and (b) the spatial variability for rainfall extremes (blue) with the black line representing the linear trend. The respective Mann-Kendall trend and associated p values are individually presented for each panel. (TIF) [file pone.0158670.s002.tif]

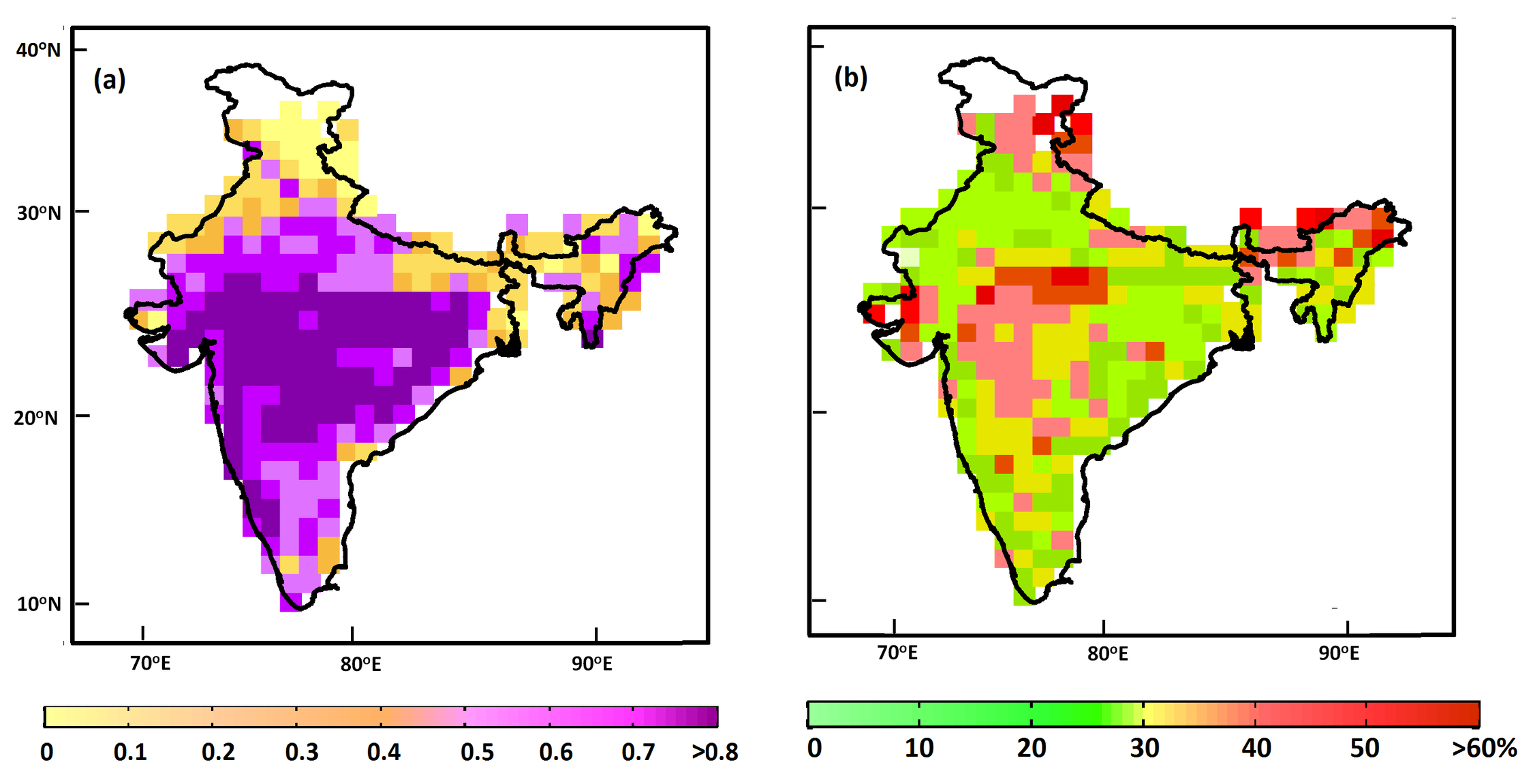

Supplement: S3 Fig — (a) Correlation between the simulated and observed soil moisture from 1979–2004. (b) Normalized Root Mean Square Error (nRMSE) for the same period. (TIF) [file pone.0158670.s003.tif]

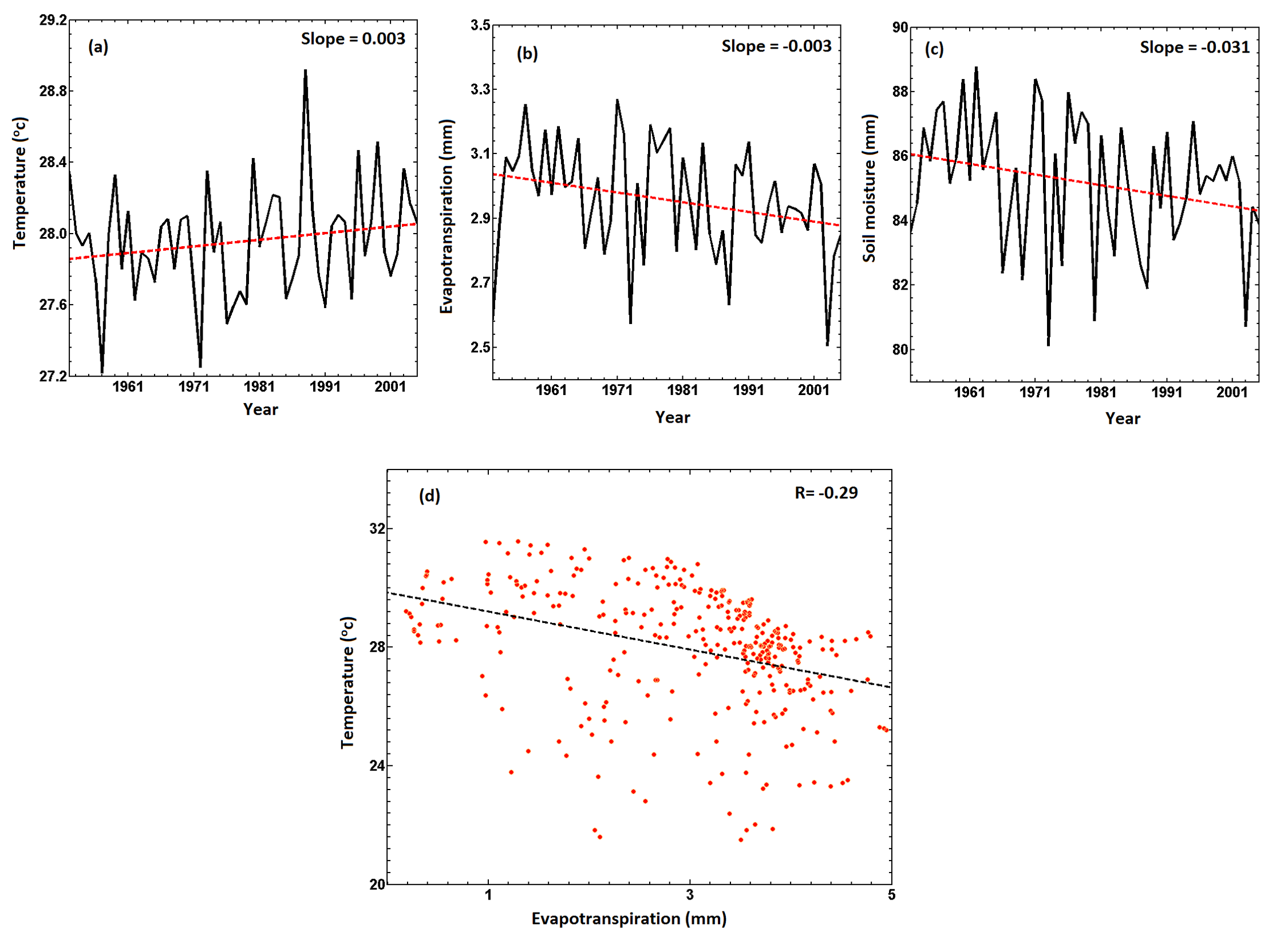

Supplement: S4 Fig — Trends in temperature (a), ET (b) and soil moisture (c) over India during 1951–2004, respectively. (d) shows a scatter plot of ET and temperature over India. The values inset the figures represent magnitude of the correlation (R) and slope for the respective variables. All the values are significant at 10% significance level. (TIF) [file pone.0158670.s004.tif]

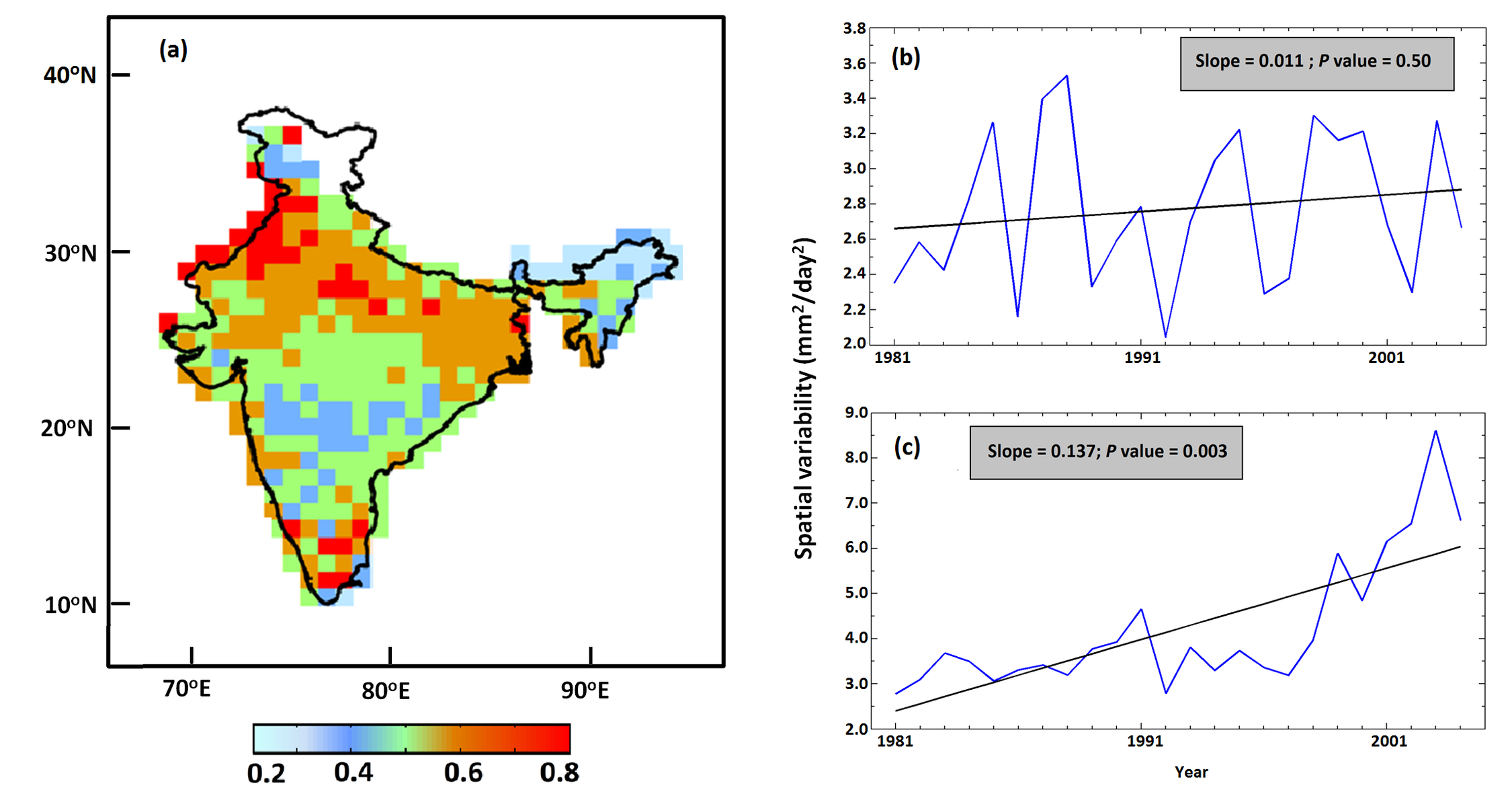

Supplement: S5 Fig — (a) Ratio of convective to total precipitation obtained from TRMM for all of India. (b) Trend in the spatial variability of the Indian Summer Monsoon Rainfall (ISMR; June-September) for the stratiform precipitation obtained from MERRA database over the Indian region (blue), where the black line indicates the fitted trend (linear) line. The Modified Mann-Kendall trend is insignificant at the 5% level. (c) is similar to (b) except for the trend in convective precipitation, which is significant at the 5% level. The spatial data obtained from TRMM and MERRA were re-gridded to IMD at a 1° resolution using a linearly interpolated technique. (TIF) [file pone.0158670.s005.tif]

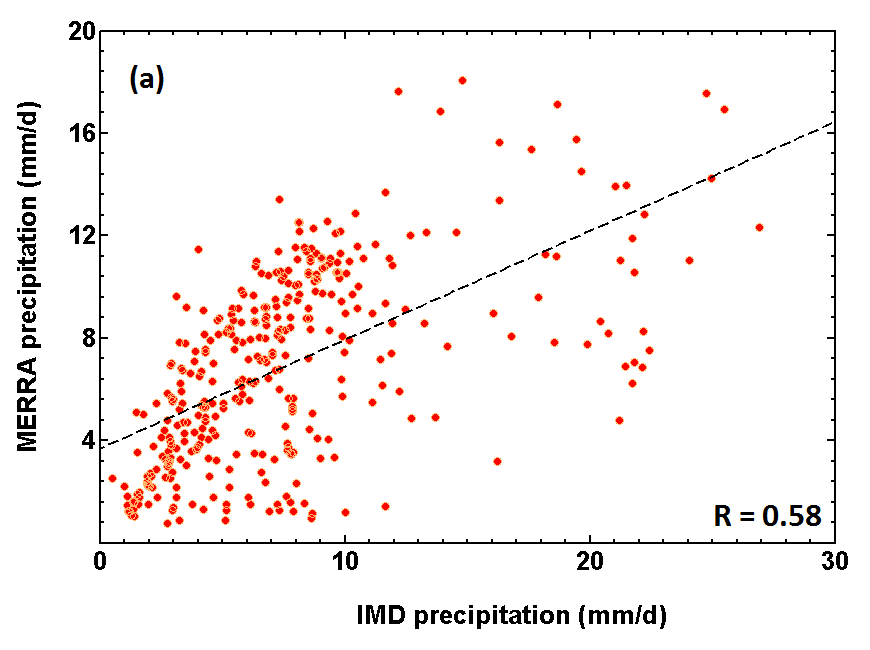

Supplement: S6 Fig — (TIF) [file pone.0158670.s006.tif]

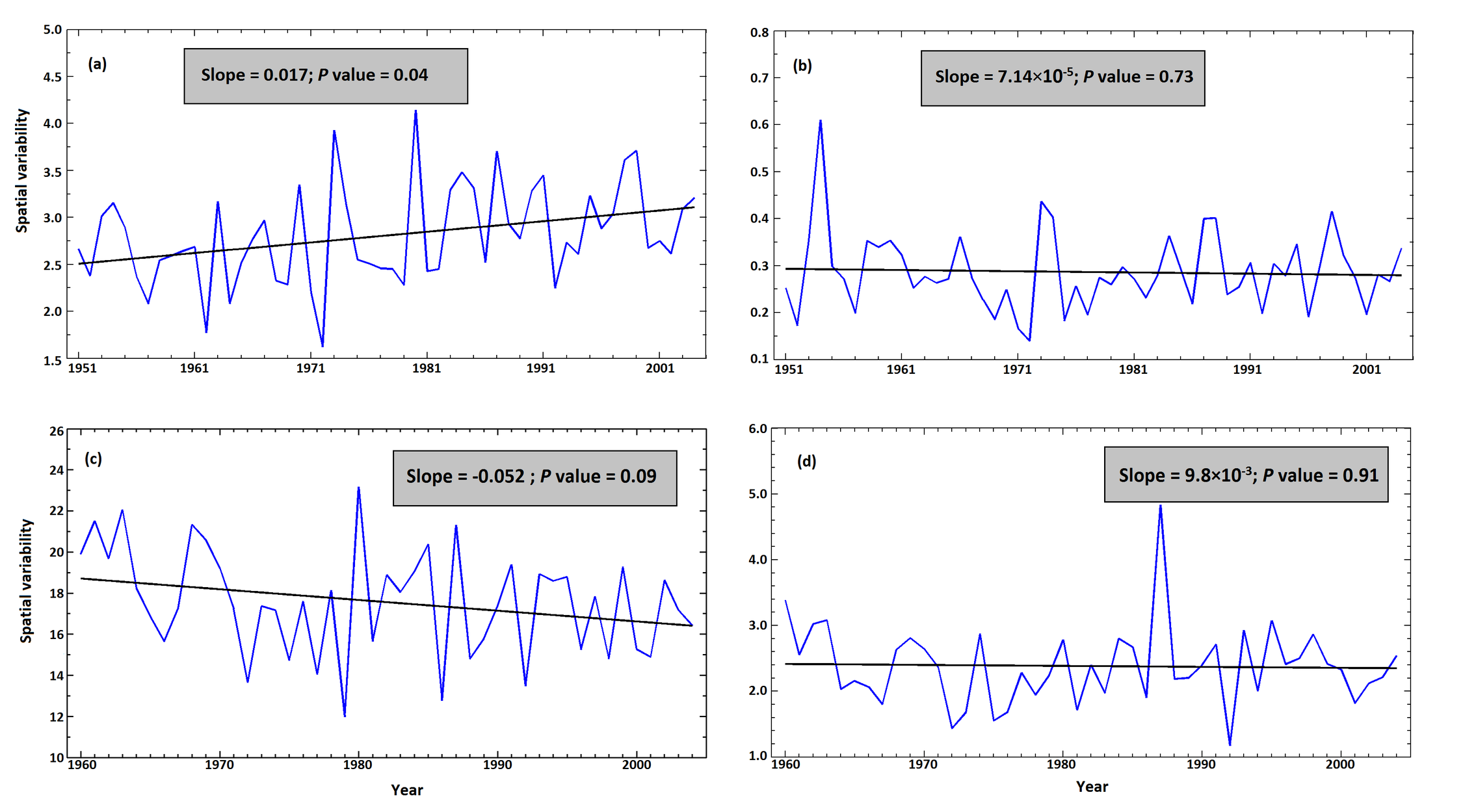

Supplement: S7 Fig — (a) The trend in the spatial variability of convective precipitation (blue) during 1951–2004 for ERA-20c reanalysis data, where the black line indicates the fitted trend (linear) line. The modified Mann-Kendall trend is significant at the 5% level. (b) is similar to (a) but shows stratiform precipitation, for which the trend is insignificant at the 5% level. Trends in convective precipitation (c) and stratiform precipitation (d) during 1961–2004 for JRA-55 reanalysis data. The modified Mann-Kendall trend is insignificant at the 5% level for both convective and stratiform precipitation. The spatial data obtained from ERA-20c and JRA-55 were regridded to a 1° resolution (same as the IMD data resolution) using a linearly interpolated technique. (TIF) [file pone.0158670.s007.tif]

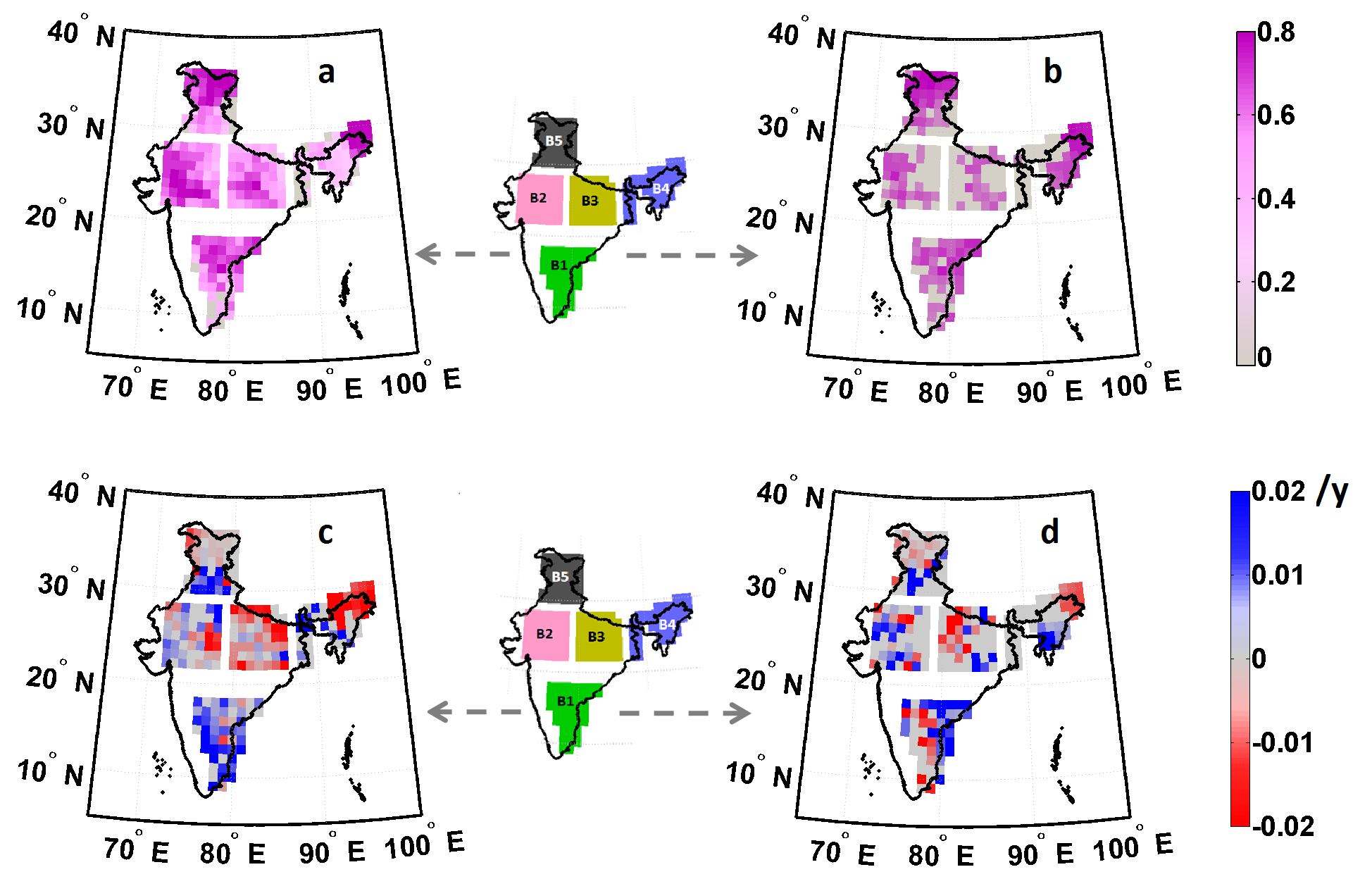

Supplement: S8 Fig — (a) Spatial correlation for the monsoonal mean and (b) spatial correlation for the seasonal maxima. (c) The trend in the spatial correlation of individual grid points over the given region estimated with a 30-year overlapping moving window for the monsoonal mean. (d) same as (c) but for seasonal maxima. (TIF) [file pone.0158670.s008.tif]

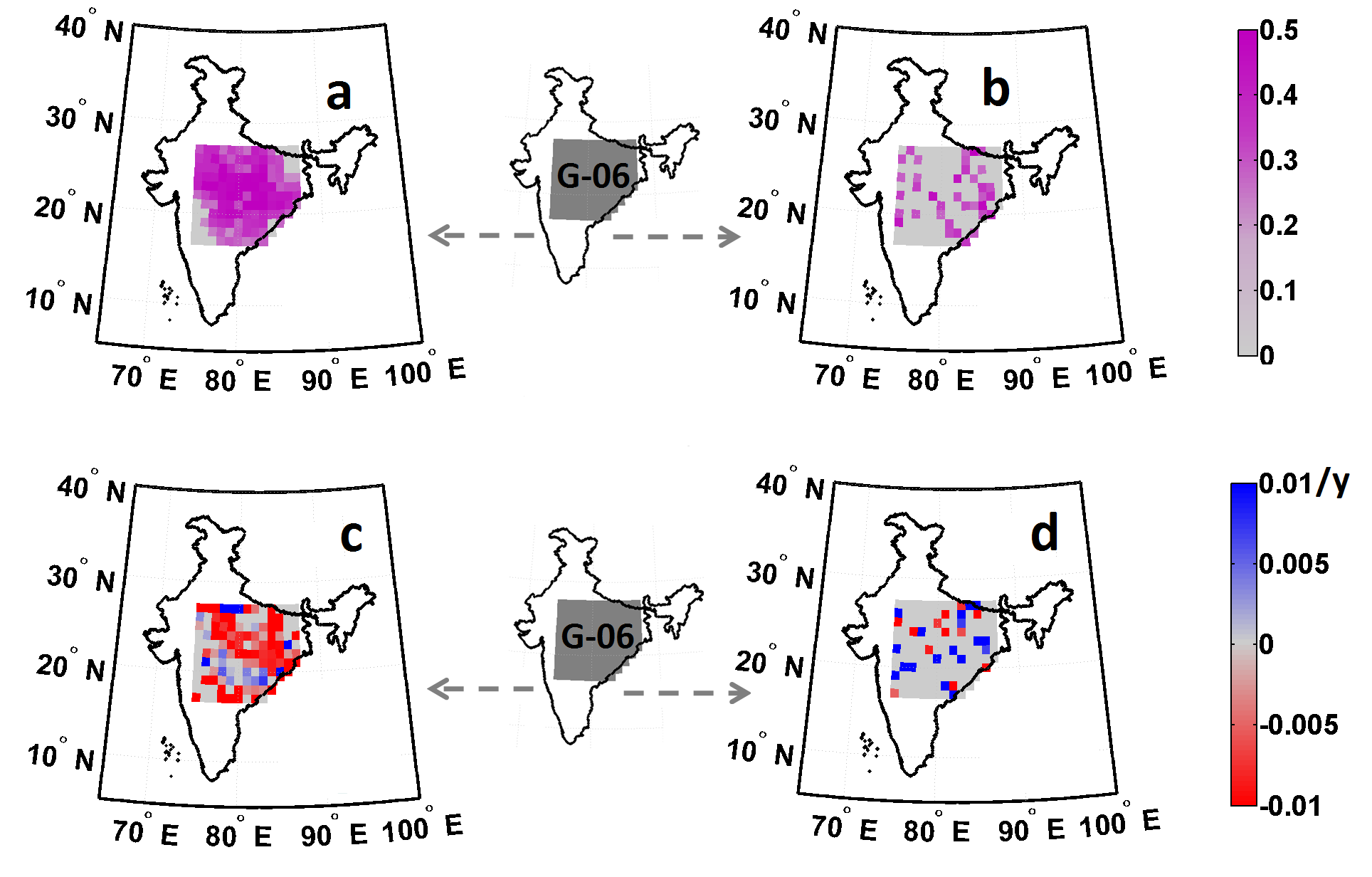

Supplement: S9 Fig — (TIF) [file pone.0158670.s009.tif]
