# Supplementary material for: Indian Summer Monsoon Rainfall: Implications of Contrasting Trends in the Spatial Variability of Means and Extremes
Source: PLoS One. 2016 Jul 27;11(7):e0158670. doi: 10.1371/journal.pone.0158670 (PMC4963102; doi:10.1371/journal.pone.0158670)
Supplement: S1 Text — Contains complete description of methods and also the Tables. (DOCX) [file pone.0158670.s010.docx]

**Supplementary material for**

**Indian Summer Monsoon Rainfall: Implications of Contrasting Trends in Spatial Variability of Means and Extremes**

Subimal Ghosh^1,2^, H. Vittal^3^, Tarul Sharma^2^, Subhankar Karmakar^2,3^, K. S. Kasiviswanathan^4^, Y. Dhanesh^4^, K. P. Sudheer^4^, and S. S. Gunthe^4,*^

^1^Dept of Civil Engineering, Indian Institute of Technology Bombay, Mumbai 400076, India

^2^Interdisciplinary Program in Climate Studies, Indian Institute of Technology Bombay, Mumbai 400 076, India

^3^Centre for Environmental Science and Engineering, Indian Institute of Technology Bombay, Mumbai 400 076, India

^4^EWRE Division, Dept of Civil Engineering, Indian Institute of Technology Madras, Chennai

^*^To whom correspondence should be made: S. S. Gunthe ([s.gunthe@iitm.ac.in](mailto:s.gunthe@iitm.ac.in))

# Methods

**Rainfall Data and Characterizing Extremes**

The gridded daily rainfall data (mm/day) at a resolution of 1° from 1901-2004 were procured from the India Meteorological Department (IMD) [1]. These rainfall data were generated with the interpolation method proposed by Shepard [2] from a fixed network of 1384 stations that had a minimum data availability of 70% to minimize the risk of generating temporal inhomogeneities. The resultant data have been previously used in many Indian monsoon studies [3, 4]*.* The convective and stratiform rainfall data were obtained from high-resolution reanalysis datasets, i.e., from the Modern-Era Retrospective Analysis for Research and Application (MERRA, resolution of 0.5° × 0.667°) database [5]*,* ERA-20C (ECMWF's first atmospheric reanalysis of the 20th century, resolution of 1° × 1°) [6], and JRA 55 (the Japanese 55-year Reanalysis, resolution of 1.25° × 1.25°) [7]. All reanalysis datasets were regridded to a IMD 1° resolution using a linearly interpolated technique.

Extreme rainfall was characterized with the block maxima method of the Extreme Value Theory (EVT) [8]; specifically, seasonal (monsoon) maxima were obtained first and then fitted to a Generalized Extreme Value (GEV) distribution. The 50-year return level was computed from the Cumulative Distribution Function (CDF) and defined as extreme according to Ghosh et al. [9]. The details of GEV distributions are explained below:

Suppose 'x' represents the annual maxima of daily precipitation in a given series, then the GEV distribution is defined by [10, 11];

$F\left( x;\mu,\alpha,\xi\right)=\left\{ \begin{aligned} exp\left\{ -\left[ 1-\frac{\xi\left( x-\mu\right)}{\alpha} \right]^{-\frac{1}{\xi}} \right\} \\ 1+\xi\left( x-\mu\right)/\alpha>0 \xi\neq0 \\ exp\left\{ -exp\left[ -\frac{\left( x-\mu\right)}{\sigma} \right] \right\} \xi=0 \end{aligned} \right.$ (1)

where μ is the location parameter, σ ˃ 0 is scale parameter and ξ is shape parameter. Depending on the shape parameter, GEV has three special cases, pointedly, the Gumbel (ξ = 0), Frechet (ξ > 0) and Weibull (ξ < 0) distributions. Further, the p year return level [which represents the (1/p) % probability of exceedance] is obtained by inverting the distribution function of the GEV (Eq. 1);

$F^{-1}\left( 1-p;\mu,\alpha,\xi\right)=\left\{ \begin{aligned} \mu-\left( \alpha/\xi\right)\left\{ 1-\left[ -\ln\left( 1-p \right) \right]^{-\xi} \right\} \xi\neq0 \\ \mu-\alpha ln\left[ -ln\left( 1-p \right) \right] \xi=0 \end{aligned} \right.$ (2)

The goodness-of-fit was evaluated using the Kolmogorov-Smirnov (KS) test. A nonparametric Gaussian kernel was fitted to the grid points instead of the GEV if the KS test failed.

**Computing Trends in the Spatial Variability**

To obtain the trend in the spatial variability of the mean monsoon rainfall, we first computed its spatial variability for a 30-year moving window with the Modified Mann-Kendall approach [12]**,** which considers both linear and nonlinear trends and is applicable to autocorrelated data. We observed high autocorrelation in the spatial variability of the mean monsoon rainfall because of smoothening with a moving average [13], and therefore, the modified Mann-Kendall approach [12,14] was implemented. This approach differs from the conventional Mann-Kendall trend in terms of the significance level. It utilizes the effective number of degrees of freedom (N_s_) adjusted for autocorrelation, rather than the total number of observations (N), to obtain the significance level. In this approach, to remove the effect of autocorrelation, a correction factor (N/N_s_) is multiplied to the variance (V) obtained from original Mann-Kendall test [12];

$V= \frac{N\left( N-1 \right)\left( 2N+5 \right)}{18}\cdot\frac{N}{N_{s}}$ (3)

$\frac{N}{N_{s}}=1+\frac{2}{N\left( N-1 \right)\left( N-2 \right)} \cdot\sum_{i=1}^{N-1} \left( N-i \right)\left( N-i-1 \right)\left( N-i-2 \right)\rho_{s}(i)$ (4)

where ρ_s_(i) is the autocorrelation functions of the ranks of the observations.

We computed the spatial variability of both the mean and extremes with a 30-year moving window throughout the entire time period used in this study. For extremes, the 50-year return levels were computed for each 30-year window, and the spatial variability was then computed. We identified significant increases and decreases in the spatial variability for the extreme and mean, respectively, after 1950, and these trends were absent prior to 1950 (Fig. S1).

We conducted the same analysis with a finer resolution for a longer-period dataset (from the same source as the 1° resolution data from IMD), which is available for the years 1901-2013 at a resolution of 0.25° [15]. We identified significant increase and decrease in the spatial variability of the extremes and mean (Fig. S2), which is consistent with the conclusion derived from Fig 1. However, for the entire observational period, the number of operating stations used to generate the gridded product was significantly inconsistent. The average number of stations per year available for data preparation was 2600, but this number varied from 1450 in the year 1901 to approximately 3950 in the period 1991 to 1994 [15]*.* This variation in the number of measurement stations may result inconsistency in the produced data, which may further introduce artifacts into the trends in the spatial variability of the mean and extremes. Hence, we continued our analysis with the 1° resolution gridded rainfall Indian monsoon data, for which the number of station was consistent [1]*.*

**Hydrological Simulations with a Variable Infiltration Capacity (VIC) Model**

The Variable Infiltration Capacity (VIC) model is a meso-scale hydrological model, first developed by [16]*,* which represents surface and subsurface hydrologic processes for spatially distributed grid cells. It can solve full water and energy balances. The VIC model is distinguished from any other hydrological models by its sub-grid parameterization of topography, soil and vegetation data. The basic features of the VIC model are as follows: 1) the land surface is modeled as a grid of large (>1 km), flat and uniform cells; 2) the inputs are time series of daily or sub-daily meteorological drivers (e.g., precipitation, air temperature, and wind speed); 3) the land-atmosphere fluxes as well as the water and energy balances at the land surface are simulated at a daily or sub-daily time step; and 4) water can only enter a grid cell via the atmosphere. Detailed information regarding the VIC model and the data requirements to simulate the hydrologic parameters can be obtained from Gao et al. [17] and Nijssen et al. [18]*.* The VIC model has been implemented for hydrological studies at the global [18, 19]*,* country [20] and watershed scales*.* In this study, the VIC model version 4.1.2 was employed in energy balance mode, which has been used to simulate the water runoff and base flow over India.

In the present study, the VIC model was run for all of India at a spatial resolution of 1° on a daily scale for the period 1951-2004; the daily meteorological data (precipitation, minimum and maximum temperature and wind speed), soil type, vegetation and topographical data served as input parameters. The precipitation, daily minimum and maximum temperature at a resolution of 1° were obtained from IMD [1,21]. The wind speed data at 1° were procured from Sheffield et al*.* [22], and these data are widely used for land surface modeling. The vegetation types were procured from Advanced Very High Resolution Radiometer (AVHRR) based on a 1-km global land classification from Hansen et al. [23], which features thirteen land cover classes along with a water class. The Leaf Area Index (LAI) considered in this study was based on Myneni et al. [24]. The information on soil parameters in India, such as the initial soil moisture content, soil layer depth, soil particle density, bulk density, elevation, etc., were obtained from the global VIC input parameters available at the VIC model website. The input files for the VIC model were prepared with these data following the method available from the VIC model website ([www.hydro.washington.edu/Lettenmaier/Models/VIC/](http://www.hydro.washington.edu/Lettenmaier/Models/VIC/)).

The VIC model output was validated with the observed soil moisture data obtained from the Essential Climate Variable - Climate Change Initiative (ESA-CCI) soil moisture product that has been developed in the framework of the European Space Agency's Water Cycle Multi-mission Observation Strategy and Climate Change Initiative projects. This product blends the soil moisture retrieval from four passive and two active microwave coarse resolution microwave sensors into a global dataset from 1979-2010 [25]. The passive microwave products were derived from Nimbus 7 Scanning Multi-channel Microwave Radiometer (SMMR), the Special Sensor Microwave Imagers (SSM/I) of the Defense Meteorological Satellite Program (DMSP), the Tropical Rainfall Measuring Mission (TRMM) and the Advanced Microwave Scanning Radiometer-Earth Observing System (AMSR-E) sensor on NASA's Aqua satellite. The active products are based on the measurements from the European Remote Sensing Satellites (ERS) 1 and 2 Active Microwave Instrument (AMI) wind scatterometer and the Advanced SCATerometer (ASCAT) from the Meteorological Operational Satellite (MetOP). Dorigo et al. [2015] indicated that this product is in good agreement with the ESA-CCI blended soil moisture data and Noah model-simulated soil moisture available from the Global Land Data Assimilation Systems (GLDAS). The detailed information regarding this product can be obtained from [www.esa-soilmoisture-cci.org/node/1](http://www.esa-soilmoisture-cci.org/node/1) and Dorigo et al. [26].

We validated the VIC simulated soil moisture with the observed soil moisture based on their correlation and normalized Root Mean Square Error (nRMSE) from 1979-2004. The nRMSE is defined as the ratio of the root mean square error and observed standard deviation [27]. The volumetric observed soil moisture data were retrieved from the satellite image for a topsoil depth of 1 cm; however, the VIC model simulates the soil moisture for the top 30 cm of soil. We assumed that the soil moisture is uniformly distributed over this shallow depth of soil and computed the simulated soil moisture for the top 1 cm. The results of the VIC-simulated soil moisture and the observed soil moisture were consistent, both in terms of the correlation and the nRMSE (Fig. S3). The magnitude of the correlation analysis were evaluated at 5% significance level and the nRMSE values were less than 20% in most part of the country.

The hydrological simulations used here may be considered an idealized run that lacks calibration but is forced with observed rainfall, temperature and wind velocity and includes observed present land use, land cover and soil properties. The purpose of this simulation is to strengthen the conclusions derived from precipitation data. The literature [28] on the calibrated VIC simulations for the Indian river basin is limited because it utilizes controlled streamflow data (river flow at downstream of reservoir) for training without considering the control structures, such as reservoir operations, or human interventions in terms of irrigation. Because naturalized streamflow data are not available, we avoided calibration in this study and conducted the analysis with the parameter values provided by Gao et al. [17] and Nijssen et al. [18]. The limitations of the hydrological simulations presented here are listed below:

1. The water yield is sensitive to land surface parameters related to soil and vegetation. Here, we only considered rice as an agricultural crop, but the cropping systems in India are far more complex and feature multiple crops, such as rice, wheat, and cereals, which differ in phenology and leaf area index.
2. A significant portion of the country is under irrigation, and in many river basins, more than 75% of agriculture depends on irrigation. Therefore, properly representing irrigation in the VIC model simulations is crucial to estimate water yields. However, because of a lack of irrigation data, we presented our hydrological experiments under natural conditions.

The above-mentioned limitations are difficult to consider because hydrologic data are lacking. Hence, we performed VIC simulations as an experimental hydrologic run. To validate the results obtained from this VIC simulation, we also conducted the analysis with precipitation. The overall results corroborate with the results obtained from the VIC simulation

**Supplementary References**

1. Rajeevan M, Bhate J, Jaswal AK. Analysis of variability and trends of extreme rainfall events over India using 104 years of gridded daily rainfall data. Geophysical research letters. 2008 Sep 1;35(18). doi:10.1029/2008GL035143.
2. Shepard D. A two-dimensional interpolation function for irregularly-spaced data. In Proceedings of the 1968 23rd ACM national conference 1968 Jan 1 (pp. 517-524). ACM.
3. Vittal H, Karmakar S, Ghosh S. Diametric changes in trends and patterns of extreme rainfall over India from pre‐1950 to post‐1950. Geophysical Research Letters. 2013 Jun 28;40(12):3253-3258. doi:10.1002/grl.50631.
4. Joshi MK, Rai A. Combined interplay of the Atlantic multidecadal oscillation and the interdecadal Pacific oscillation on rainfall and its extremes over Indian subcontinent. Climate Dynamics. 2014 Sep;44(11-12):3339-3359.
5. Rienecker MM, Suarez MJ, Gelaro R, Todling R, Bacmeister J, Liu E, et al. MERRA: NASA's modern-era retrospective analysis for research and applications. Journal of Climate. 2011 Jul;24(14):3624-3648.
6. Poli P, Hersbach H, Tan D, Dee D, Thepaut JN, Simmons A, et al. The data assimilation system and initial performance evaluation of the ECMWF pilot reanalysis of the 20th-century assimilating surface observations only (ERA-20C). 2013.
7. Kobayashi S, Ota Y, Harada Y, Ebita A, Moriya M, Onoda H, et al. The JRA-55 reanalysis: General specifications and basic characteristics. [Journal of the Meteorological Society of Japan](https://www.jstage.jst.go.jp/browse/jmsj). 2015;93(1):5-48.
8. Furrer EM, Katz RW, Walter MD, Furrer R. Statistical modeling of hot spells and heat waves. Climate research (Open Access for articles 4 years old and older). 2010 Oct 19;43(3):191.
9. Ghosh S, Das D, Kao SC, Ganguly AR. Lack of uniform trends but increasing spatial variability in observed Indian rainfall extremes. Nature Climate Change. 2012 Feb 1;2(2):86-91.
10. Coles S. An introduction to statistical modeling of extreme values. London: Springer; 2001 Dec.
11. Katz RW, Parlange MB, Naveau P. Statistics of extremes in hydrology. Advances in water resources. 2002 Dec 31;25(8):1287-1304.
12. Hamed KH, Rao AR. A modified Mann-Kendall trend test for autocorrelated data. Journal of Hydrology. 1998 Jan 30;204(1):182-196.
13. McCabe GJ, Palecki MA, Betancourt JL. Pacific and Atlantic Ocean influences on multidecadal drought frequency in the United States. Proceedings of the National Academy of Sciences. 2004 Mar 23;101(12):4136-4141.
14. Kendall MG. Rank correlation methods. London :Griffin; 1955.
15. Pai, D. S. et al. Development and analysis of a new high spatial resolution (0.250× 0.250) long period (1901–2010) daily gridded rainfall data set over India" (Tech. Rep. 1/2013, National Climate Centre Research, India Meteorological Department, Pune, 2013).
16. Liang X. A two-layer variable infiltration capacity land surface representation for general circulation models. 1994. Series, TR140, 208 pp., Univ. of Washington, Seattle.
17. Gao H, Tang Q, Shi X, Zhu C, Bohn T, Su F, et al. Water budget record from variable infiltration capacity (VIC) model algorithm theoretical basis document. Dept. Civil and Environmental Eng., Univ. Washington, Seattle, WA. 2009:09-18.
18. Nijssen B, O'Donnell GM, Lettenmaier DP, Lohmann D, Wood EF. Predicting the discharge of global rivers. Journal of Climate. 2001 Aug;14(15):3307-23.
19. Nijssen B, Schnur R, Lettenmaier DP. Global retrospective estimation of soil moisture using the variable infiltration capacity land surface model, 1980-93. Journal of Climate. 2001 Apr;14(8):1790-808.
20. Maurer EP, Wood AW, Adam JC, Lettenmaier DP, Nijssen B. A long-term hydrologically based dataset of land surface fluxes and states for the conterminous United States. Journal of climate. 2002 Nov;15(22):3237-51.
21. Srivastava AK, Rajeevan M, Kshirsagar SR. Development of a high resolution daily gridded temperature data set (1969–2005) for the Indian region. Atmospheric Science Letters. 2009 Oct 1;10(4):249-54.
22. Sheffield J, Goteti G, Wood EF. Development of a 50-year high-resolution global dataset of meteorological forcings for land surface modeling. Journal of Climate. 2006 Jul;19(13):3088-111.
23. Hansen MC, DeFries RS, Townshend JR, Sohlberg R. Global land cover classification at 1 km spatial resolution using a classification tree approach. International journal of remote sensing. 2000 Jan 1;21(6-7):1331-64.
24. Myneni RB, Ramakrishna R, Nemani R, Running SW. Estimation of global leaf area index and absorbed PAR using radiative transfer models. Geoscience and Remote Sensing, IEEE Transactions on. 1997 Nov;35(6):1380-93.
25. Dorigo WA, Gruber A, De Jeu RA, Wagner W, Stacke T, Loew A, et al. Evaluation of the ESA CCI soil moisture product using ground-based observations. Remote Sensing of Environment. 2015 Jun 1;162:380-95.
26. Dorigo W, Jeu R, Chung D, Parinussa R, Liu Y, Wagner W, Fernández‐Prieto D. Evaluating global trends (1988–2010) in harmonized multi‐satellite surface soil moisture. Geophysical Research Letters. 2012 Sep 1;39(18).
27. Li J, Islam S. Estimation of root zone soil moisture and surface fluxes partitioning using near surface soil moisture measurements. Journal of Hydrology. 2002 Mar 1;259(1):1-4.
28. Raje D, Krishnan R. Bayesian parameter uncertainty modeling in a macroscale hydrologic model and its impact on Indian river basin hydrology under climate change. Water Resources Research. 2012 Aug 1;48(8).

**List of Tables**

**Table A.** The changes in the mean, 25th percentile, median and 75th percentile for all major river basins in India. The water yield data for the respective basins were separated for two different periods (1950-1975 and 1976-2000) to assess the possible shift in the temporal pattern of the water yield.

| **Basins** | **Changes(%) in Recent Period (1976-2000) with respect to past (1951-1975)** | | | |
| --- | --- | --- | --- | --- |
|  | **Mean** | **25th percentile** | **Median** | **75th percentile** |
| **Deficit basins** | | | | |
| Indus | 11.41 | 42.81 | 13.70 | -2.21 |
| Ganga | 7.84 | 8.08 | 10.42 | 0.076 |
| East flow river –II | 14.71 | 5.96 | 8.68 | 23.29 |
| Narmada | 0.48 | 2.42 | -3.28 | -1.32 |
| West flow river- II | 7.88 | 38.14 | 24.80 | 16.54 |
| Tapti | 1.69 | -10.32 | -12.95 | -3.27 |
| Yamuna | -4.82 | 2.86 | 0.97 | -14.91 |
| Cauvery | -1.94 | -7.32 | 2.24 | 2.18 |
| Krishna | -9.47 | -8.57 | -7.72 | -9.33 |
| **Surplus basins** | | | | |
| Mahanadi | -11.74 | 2.95 | -13.17 | -20.59 |
| Godavari | -1.30 | 3.73 | -2.49 | -9.00 |
| West flow river –I | -10.06 | -9.23 | -4.23 | -11.69 |
| East flow river-I | -3.95 | -5.17 | -7.38 | -1.83 |
| Brahmari | -4.89 | -3.78 | -7.69 | -0.0057 |
| Brahmaputra | 8.90 | 8.23 | 6.43 | 11.08 |
| Mahi | 17.51 | 55.57 | 43.10 | 35.28 |
| Megna | 44.91 | 0.007 | 21.55 | 49.37 |

**Table B.** Extent of regions considered in the study.

| **Box** | **Lat (^o^N)** | **Lon (^o^E)** |
| --- | --- | --- |
| B1 | 8.5 to 17.5 | 75.5 to 82.5 |
| B2 | 21.5 to 27.5 | 71.5 to 77.5 |
| B3 | 21.5 to 27.5 | 79.5 to 85.5 |
| B4 | 21.5 to 29.5 | 87.5 to 96.5 |
| B5 | 29.5 to 35.5 | 73.5 to 79.5 |
| G06 | 16.6 to 26.5 | 74.5 to 86.5 |

**Table C.** The calculated arithmetic mean and derived variance as well as the relative difference (%) for the long-term ISMR (1951-2004). The total climatological data were separated into two different periods (1950-1975 and 1976-2000) to assess and verify the possible shift in the spatial pattern of monsoon rainfall. The table shows the arithmetic mean and variance for all India, five different regions, and the Central Indian region, as adopted by Goswami et al. [2006].

| **Parameters** | **Mean monsoon** | | | | **Seasonal maxima (50 yr RL)** | | |
| --- | --- | --- | --- | --- | --- | --- | --- |
|  | ***1950-1975*** | | ***1976-2000*** | ***% change*** | ***1950-1975*** | ***1976-2000*** | ***% change*** |
| **All India** | | | | | | | |
| Mean (mm/d) | 7.856 | 7.860 | | 0.05 | 184.4 | 232.6 | 26.13 |
| Variance (mm^2^/d^2^) | 29.16 | 26.84 | | -7.95 | 6776.9 | 18469.1 | 172.53 |
| **B1** | | | | | | | |
| Mean (mm/d) | 3.509 | 3.588 | | 2.25 | 112.42 | 125.44 | 11.58 |
| Variance (mm^2^/d^2^) | 2.182 | 2.002 | | -8.24 | 2029.51 | 2237.63 | 10.25 |
| **B2** | | | | | | | |
| Mean (mm/d) | 5.292 | 5.151 | | -2.66 | 195.67 | 212.86 | 8.78 |
| Variance (mm^2^/d^2^) | 4.413 | 4.307 | | -2.40 | 4350.28 | 8192.7 | 88.35 |
| **B3** | | | | | | | |
| Mean (mm/d) | 8.301 | 8.165 | | -1.63 | 181.623 | 227.67 | 25.35 |
| Variance (mm^2^/d^2^) | 2.121 | 1.902 | | -10.32 | 1742.24 | 5126.77 | 194.26 |
| **B4** | | | | | | | |
| Mean (mm/d) | 13.515 | 14.319 | | 5.94 | 255.066 | 337.254 | 32.22 |
| Variance (mm^2^/d^2^) | 36.96 | 25.54 | | -30.89 | 15689.57 | 7293.47 | -53.51 |
| **B5** | | | | | | | |
| Mean (mm/d) | 6.353 | 6.508 | | 2.43 | 167.05 | 214.97 | 28.68 |
| Variance (mm2/d2) | 8.676 | 9.467 | | 9.11 | 3363.51 | 6930.47 | 106.04 |
| **G06** | | | | | | | |
| Mean (mm/d) | 7.197 | 7.07 | | -1.76 | 179.64 | 208.43 | 16.02 |
| Varience(mm^2^/d^2^) | 4.044 | 3.760 | | -7.02 | 3433.57 | 6606.36 | 92.40 |
